# Evolutionary Changes in the Interaction of miRNA With mRNA of Candidate Genes for Parkinson’s Disease

**DOI:** 10.3389/fgene.2021.647288

**Published:** 2021-03-30

**Authors:** Saltanat Kamenova, Assel Aralbayeva, Aida Kondybayeva, Aigul Akimniyazova, Anna Pyrkova, Anatoliy Ivashchenko

**Affiliations:** ^1^Faculty of Medicine and Health Care, Al-Farabi Kazakh National University, Almaty, Kazakhstan; ^2^Department of Neurology, Kazakh Medical University, Almaty, Kazakhstan; ^3^Faculty of Biology and Biotechnology, Al-Farabi Kazakh National University, Almaty, Kazakhstan

**Keywords:** gene, phylogeny, Parkinson’s disease, miRNA, mRNA, association, marker

## Abstract

Parkinson’s disease (PD) exhibits the second-highest rate of mortality among neurodegenerative diseases. PD is difficult to diagnose and treat due to its polygenic nature. In recent years, numerous studies have established a correlation between this disease and miRNA expression; however, it remains necessary to determine the quantitative characteristics of the interactions between miRNAs and their target genes. In this study, using novel bioinformatics approaches, the quantitative characteristics of the interactions between miRNAs and the mRNAs of candidate PD genes were established. Of the 6,756 miRNAs studied, more than one hundred efficiently bound to mRNA of 61 candidate PD genes. The miRNA binding sites (BS) were located in the 5′-untranslated region (5′UTR), coding sequence (CDS) and 3′-untranslated region (3′UTR) of the mRNAs. In the mRNAs of many genes, the locations of miRNA BS with overlapping nucleotide sequences (clusters) were identified. Such clusters substantially reduced the proportion of nucleotide sequences of miRNA BS in the 5′UTRs, CDSs, and 3′UTRs. The organization of miRNA BS into clusters leads to competition among miRNAs to bind mRNAs. Differences in the binding characteristics of miRNAs to the mRNAs of genes expressed at different rates were identified. Single miRNA BS, polysites for the binding for one miRNA, and multiple BS for two or more miRNAs in one mRNA were identified. Evolutionary changes in the BS of miRNAs and their clusters in 5′UTRs, CDSs and 3′UTRs of mRNA of orthologous candidate PD genes were established. Based on the quantitative characteristics of the interactions between miRNAs and mRNAs candidate PD genes, several associations recommended as markers for the diagnosis of PD.

## Introduction

Parkinson’s disease (PD) is a neurodegenerative disease with a high mortality rate (Sadlon et al., 2019; Zhang et al., 2019; Zhao and Wang, 2019; Salamon et al., 2020). The development of the disease occurs over several years, which raises the possibility that diagnostic methods may be developed to facilitate subsequent therapy. Unfortunately, at present, there are no effective methods for the early diagnosis of this disease, which significantly reduces the effects of treatment (Arshad et al., 2017; Patil et al., 2019). The difficulty of diagnosing PD is attributable to the many genes that participate in the development of this disease (candidate genes), the expression of which changes with the development of several types of neurodegenerative diseases (Behbahanipour et al., 2019). To date, several dozen candidate PD genes have been identified, and their roles in PD must be investigated. Some of these genes are candidate genes for Alzheimer’s disease, PD, dementia, Huntington’s disease, frontotemporal dementia, and other neurodegenerative diseases (Brennan et al., 2019; Dong et al., 2020; Yan et al., 2020). Several dozen genes encode proteins containing polyglutamine, the number of which ranges from 30 to 100 or more (Cao et al., 2017). It is believed that both the type of neurodegenerative disease and its severity are associated with the number of glutamine residues (Chen et al., 2018). Previous studies have attempted to link single nucleotide polymorphisms with the probability of PD and other neurodegenerative diseases (D’Anca et al., 2019; Hu et al., 2019; Kakati et al., 2019). Several candidate genes for neurodegenerative diseases contain miRNA binding sites (BS) encoding oligopeptides (Niyazova et al., 2015; Kondybayeva et al., 2018) that are believed to be responsible for the development of Alzheimer’s disease, PD, dementia, and Huntington’s disease. Therefore, it is necessary to establish which miRNAs can interact in such mRNA regions. In recent years, there has been an increased interest in miRNAs, which can selectively alter gene expression and, to varying degrees, regulate it in different tissues (Jurjević et al., 2017; Marques et al., 2017; Starhof et al., 2019; Uwatoko et al., 2019; Chen et al., 2020; Fejes et al., 2020; Nie et al., 2020; Ozdilek and Demircan, 2020; Ravanidis et al., 2020). Considering the possibility that miRNAs may be synthesized in one tissue and subsequently transferred through the bloodstream to other tissues, the issue of their regulation of the expression of candidate disease genes is complex (Ravanidis et al., 2020; Thomas et al., 2020; Wang et al., 2020; Xie et al., 2020). The human genome encodes more than seven thousand miRNAs, some of which can interact with mRNAs of several genes (Ivashchenko et al., 2014a,b,c; Niyazova et al., 2015; Atambayeva et al., 2017), and some genes are the potential targets of many miRNAs (Kondybayeva et al., 2018, 2020; Aisina et al., 2019; Mukushkina et al., 2020), which also makes it difficult to identify selective markers of the disease. The use of well-known bioinformatic approaches did not lead to the identification of reliable miRNA markers of diseases. In this work, we studied the quantitative characteristics of the interactions of known miRNAs with the mRNAs of candidate PD genes. Quantitative characteristics are a necessary and important parameter for assessing the effectiveness of the interactions between miRNAs and mRNAs. The competition among miRNAs to suppress the expression of one gene by positioning their BS with overlapping nucleotide sequences in regions of the mRNAs called clusters has been demonstrated (Aisina et al., 2019; Mukushkina et al., 2020). Additionally, it should be noted that approximately half of the miRNAs are derived from the introns of the host genes, while the rest of the miRNAs are encoded in intergenic regions (Berillo et al., 2013). Consequently, the host gene can be a source of miRNA and, at the same time, a target of miRNA. Since miRNA can be quickly transferred between tissues through the bloodstream, this characteristic considerably complicates the establishment of the origin of miRNA circulating in the blood (Chen et al., 2018; Rosas-Hernandez et al., 2018; Brennan et al., 2019; Ramaswamy et al., 2020). Therefore, it is necessary to reveal the quantitative characteristics of the interactions of all known miRNAs with candidate genes and subsequently investigate the most effective associations of miRNAs and potential target genes. This approach eliminates many artifacts and enables us to increase the reliability of establishing effective associations of miRNAs and candidate genes.

The expression of candidate genes depends on several factors, including miRNAs that regulate gene expression at the posttranscriptional stage (Leggio et al., 2017; Lu et al., 2017; Martinez and Peplow, 2017; Quinlan et al., 2017; Li et al., 2018; Liu et al., 2019; Patil et al., 2019; Qin et al., 2019). It has been established that some miRNAs can interact with several or even hundreds of genes (Atambayeva et al., 2017), and the reverse situation is also observed: one gene can be the target of many miRNAs (Niyazova et al., 2015; Kondybayeva et al., 2018; Aisina et al., 2019). These properties greatly complicate the identification of miRNA associations and genes that can serve as markers of diseases. Many researchers have studied the changes in the concentrations of several miRNAs or the manipulation of the expression levels of several genes related to PD, and based on these studies, correlations were established between the expression levels of miRNAs and genes (Recabarren and Alarcón, 2017; Ren et al., 2019). Correlation does not allow establishing a direct dependence between miRNA and potential target genes. Consequently, the identification of such correlations does not enable us to establish specific relationships between miRNAs and target genes. Therefore, after over two decades of studying miRNA, no method has been developed for diagnosing various diseases using miRNA. Given the above circumstances, using the MirTarget program, we searched for associations of known human miRNAs with candidate PD genes. To confirm the reliability of these associations, it is necessary to show their presence in orthologous genes.

It is necessary to examine the expression of candidate genes in tissues affected by diseases. The level of miRNA expression in tissues with potential target genes and the possibility of delivering miRNA *via* blood to such tissues must be determined. Many studies have shown that miRNAs can circulate in the blood as components of exosomes and can enter almost any cell (Singh and Sen, 2017; Viswambharan et al., 2017; Wang et al., 2017; Rostamian Delavar et al., 2018; Titze-de-Almeida and Titze-de-Almeida, 2018; Tolosa et al., 2018; Yang et al., 2019; Ozdilek and Demircan, 2020; Wang and Zhang, 2020). Even in one tissue, the transfer of miRNA by diffusion was less effective than the transfer of miRNAs through the bloodstream to organ tissues. Bioinformatic approaches have been employed to identify associations between miRNAs and candidate genes (Zhang et al., 2019; Zhao and Wang, 2019). Such approaches enable us to study a substantial number of options for the interactions of known human miRNAs with all human protein-encoding genes. In this paper, we searched for miRNAs that bind to candidate PD genes to establish effective associations between miRNAs and genes that may be employed for the diagnosis of PD.

## Materials and Methods

The nucleotide (nt) sequences of candidate genes of PD were downloaded from the NCBI^1^. These specific candidate genes are shown in Supplementary Table 1. The nucleotide sequences of 2,565 miRNAs (old miRNAs) were obtained from miRBase, and 3,707 miRNAs (new miRNAs) were obtained from a previous study (Londin et al., 2015). The reads per kilobase million (RPKM) value (Mortazavi et al., 2008) was provided in the Human Protein Atlas data^2^. Orthologous genes of the following objects were used in the work: *B*os *mutus* (bta), *Bubalus bubalis (bbu), Callithrix jacchus* (cja), *Capra hircus (chi), Delphinapterus leucas* (dle), *Felis catus* (fca), *Gorilla gorilla* (ggo), *Homo sapiens* (hsa), *Macaca fascicularis* (mfa), *Macaca mulatta* (mml), *Macaca nemestrina* (mne), *Mus musculus* (mmu), *Nomascus leucogenys* (nle), *Odobenus rosmarus divergens* (ord), *Orcinus orca* (oor), *Ovis aries (oar), Pongo abelii* (pab), *Papio anubis* (pab), *Pan paniscus* (ppa), *Panthera pardus* (ppr), *Pan troglodytes* (ptr), *Saimiri boliviensis* (sbo), and *Sus scrofa* (ssc). The miRNA BS in the 5′-untranslated region (5′UTR), coding sequence (CDS), and 3′-untranslated region (3′UTR) of several genes were predicted using the MirTarget program (Ivashchenko et al., 2014b, 2016). This program defines the following features of miRNA binding to mRNAs: (a) the start of the initiation of miRNA binding to mRNAs; (b) the localization of miRNA BSs in the 5′UTRs, CDSs and 3′UTRs of mRNAs; (c) the free energy of the interaction between miRNAs and mRNAs (ΔG, kJ/mole); and (d) the schemes of nucleotide interactions between miRNAs and mRNAs. The ratio ΔG/ΔGm (%) was determined for each site (ΔGm equals the free energy of miRNA binding with its fully complementary nucleotide sequence). The miRNA BSs located in mRNAs had ΔG/ΔGm ratios of 90% or more. The ΔG/ΔGm ratios were determined on the assumption that the members of one miRNA family generally differed by no more than one to three nucleotides, and along with a miRNA length of 22 nt, the ΔG/ΔGm value was determined to be 90% (20 nt/22 nt = 90%)±96% (21 nt/22 nt = 96%). With a larger difference in the number of mismatched nucleotides, the probability of two or more miRNAs binding to one site increases, despite the natural ability of miRNAs to interact selectively with the mRNAs of the target gene. The MirTarget program identifies the positions of the BSs on the mRNA, beginning with the first nucleotide of the mRNA’s 5′UTR. The MirTarget program identifies hydrogen bonds between adenine (A) and uracil (U), guanine (G) and cytosine (C), G and U, A and C. The distance between A and C was 1.04 nanometers; the distance between G and C and between A and U was 1.03 nanometers; and the distance between G and U was 1.02 nanometers (Leontis et al., 2002). The numbers of hydrogen bonds in the G-C, A-U, G-U, and A-C interactions were 3, 2, 1, and 1, respectively (Kool, 2001; Lemieux and Major, 2002; Leontis et al., 2002; Garg and Heinemann, 2018). Taking into account the formation of non-canonical pairs significantly increases the reliability of establishing the interaction of miRNAs with mRNAs. The MirTarget program determines single miRNA BSs in mRNAs and miRNA BSs in clusters (arranged in series with overlapping nucleotide sequences of the same or several miRNAs). In this study, we suppose that the miRNA BSs in mRNAs were organized into clusters, which can be used as effective PD markers.

## Results

An analysis of the interactions between miRNAs and mRNAs was performed with candidate PD genes with an RPKM expression, considering the location of miRNA BSs in the 5′UTRs, CDSs and 3′UTRs. This approach enabled us to determine which miRNAs bound to different mRNA sites and which miRNAs preferred to interact with genes with different expression levels, since the results of the interactions between miRNAs and mRNAs are dependent on the ratio of the miRNA and mRNA concentrations. For example, the strong interaction of miRNAs with mRNAs slightly inhibits translation at miRNA concentrations that are tens of times lower than the mRNA concentrations. Conversely, the average interaction of miRNA with mRNA at substantially higher concentrations of miRNA over mRNA leads to significant suppression of translation. It is important to quantify the interactions between miRNAs and mRNAs to comparatively evaluate competition among miRNAs when they bind to mRNA.

### Characteristics of the Interactions Between miRNAs and the 5′UTRs of mRNAs of Candidate PD Genes

Table 1 shows the data on the characteristics of the interactions between miRNAs and the mRNAs of the *GSK3B, PPARGC1A, ZFAND4*, and *CCNY* genes, depicting the cluster organization of the BSs of many miRNAs. The *GSK3B* gene serves as the potential target of 22 miRNAs, which distinguishes it from other candidate PD genes. The cluster of 22 miRNA BSs was located between the third and thirty-nine nucleotides (Table 1). The beginnings of these BSs were located over three nucleotides, which corresponded to their connection with the reading frame. In the mRNA of the *MANF* and other genes, paired miRNA BSs were also located over three nucleotides (Supplementary Table 2). The total length of the 22 BSs of *GSK3B* mRNA, considering multiple BSs, was 624 nt, which was 16 times greater than the length of the cluster. Gene *GSK3B* has BS for ID00296.3p-miR, ID00756.3p-miR, ID01804.3p-miR, ID02064.5p-miR with ΔG value more than −130 kJ/mole. ID01804.3p-miR, ID00457.3p-miR, ID00061.3p-miR, ID03151.3p-miR, ID02064.5p-miR, and miR-3960 have two BS, which significantly increased the effect of these miRNAs on the expression of the *GSK3B* gene.

**TABLE 1:** Characteristics of miRNA interactions with 5′UTR mRNAs of candidate PD genes.

**Gene; RPKM**	**miRNA**	**Start of site, nt**	**ΔG, kJ/mole**	**ΔG/ΔGm, %**	**Length, nt**
*GSK3B*; 8.3	ID02187.5p-miR	3	−123	89	23
	ID03229.5p-miR	4	−123	92	22
	ID01804.3p-miR	5, 12	−134	91	23
	ID00756.3p-miR	8	−123	89	23
	ID01041.5p-miR	8	−132	90	24
	ID02294.5p-miR	8	−127	87	24
	ID00457.3p-miR	8, 11	−123, −129	91, 95	22
	ID03367.5p-miR	11	−119	95	20
	ID00061.3p-miR	8,11	−125÷−136	91÷98	22
	ID00296.3p-miR	9	−138	88	25
	ID01641.3p-miR	9	−127	86	24
	ID03151.3p-miR	9, 12	−115	93	20
	ID03229.5p-miR	10	−123	92	22
	ID01702.3p-miR	13	−140	93	24
	ID02064.5p-miR	10, 13	−129, −136	90, 94	23
	ID01873.3p-miR	11	−123	94	21
	miR-3960	11, 14	−115	92	20
	ID02522.3p-miR	12	−127	91	23
	ID02499.3p-miR	13	−119	92	21
	ID02429.3p-miR	14	−125	92	23
	ID01652.3p-miR	15	−125	89	23
	ID02538.3p-miR	15	−121	90	22
*PPARGC1A*; 2.5	ID00470.5p-miR	18÷47 (5)	−108÷−110	89÷91	23
	[1.5] miR-574-5p	20÷31 (5)	−108 ÷−113	89–93	23
	ID02299.5p-miR	30	−98	92	21
	ID02732.3p-miR	36, 42	−121	89	23
	ID03332.3p-miR	71	−134	90	24
	ID01310.3p-miR	135÷144 (4)	−121÷−123	92÷94	22
	ID03332.3p-miR	143, 146	−134 −140	90, 94	24
	ID02761.3p-miR	149	−132	89	24
*ZFAND4*; 0.5	ID03418.3p-miR	109	−123	87	23
	ID00296.3p-miR	112	−134	85	25
	ID03206.5p-miR	114	−115	92	20
	ID01190.5p-miR	114	−136	89	24
	ID00030.3p-miR	114	−125	94	22
	ID02294.5p-miR	114	−125	86	24
	ID01574.5p-miR	116	−121	86	23
	ID01804.3p-miR	118	−125	86	23
	ID01702.3p-miR	118	−129	86	24
	ID03367.5p-miR	118	−113	90	20
	ID03073.3p-miR	128	−129	94	23
*CCNY*; 19.7	ID01041.5p-miR	1	−136	93	24
	ID01873.3p-miR	1	−123	94	21
	ID00296.3p-miR	4	−140	89	25
	ID01702.3p-miR	4	−134	89	24
	ID01641.3p-miR	4	−132	89	24
	ID01106.5p-miR	7	−132	89	24
	ID01879.5p-miR	8	−129	95	22
	ID02229.3p-miR	9	−121	92	21
	ID02499.3p-miR	9	−123	95	21
	ID03027.3p-miR	11	−121	85	24

*In Table 1 and below, the number of miRNA binding sites is indicated in oval brackets. The value of miRNAs RPKM is indicated in square brackets.*

**FIGURE 1:**
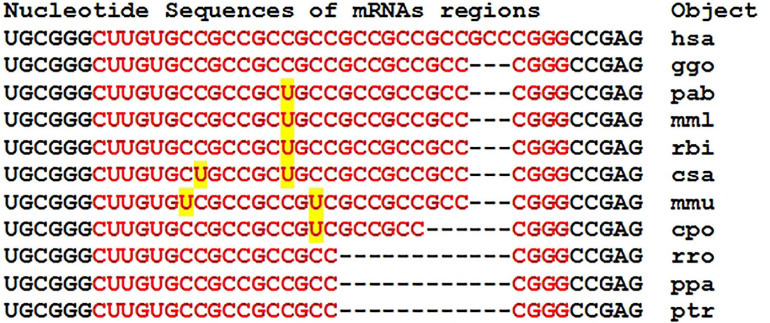
Nucleotide sequences of 5′UTR regions of mRNAs of orthologous *GSK3B* genes containing clusters of miRNAs binding sites.

Orthologous genes can be used as evidence of the reality of miRNA BS with the potential target gene mRNA. Figure 1 shows the nucleotide sequences of the BS of several miRNAs included in the mRNA cluster orthologs of the *GSK3B* gene. The obtained results show that the nucleotide sequences of the clusters decrease from 33 nt in the hsa-mRNA of the *GSK3B* gene to 22 nt in the ptr-mRNA. Therefore, starting from ptr-mRNA, the cluster contains miRNAs BS of 21 nt or more, which can bind miRNAs of orthologous genes. Note that changes in the nucleotide composition of BS occur according to the principle of replacement of purine for purine (A ↔ G), or pyrimidine for pyrimidine (U ↔ C). With such substitutions, non-canonical G-U and A-C pairs are formed, or the canonical G-C and A-U pairs are formed (Figure 1). Clusters of miRNA BS in the mRNA of all objects are located between the conserved oligonucleotides UGCGGG and CCGAG. All cluster regions in orthologous genes of *GSK3B* include the same pentanucleotide CGGGC.

**FIGURE 2:**
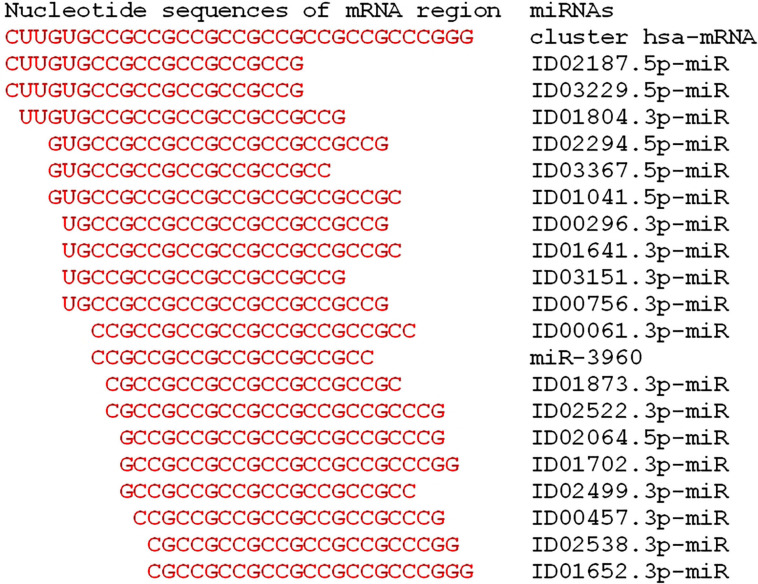
Schemes of the location of miRNAs binding sites in the cluster located in the 5′UTR of the mRNA of the candidate hsa-*GSK3B* gene of Parkinson’s disease.

Figure 2 shows the location of the miRNA BS within the cluster, which demonstrate competition between miRNAs when they bind in the mRNA cluster of the *GSK3B* gene. Binding of any of the miRNAs in the cluster interferes with the binding of other miRNAs.

**FIGURE 3:**
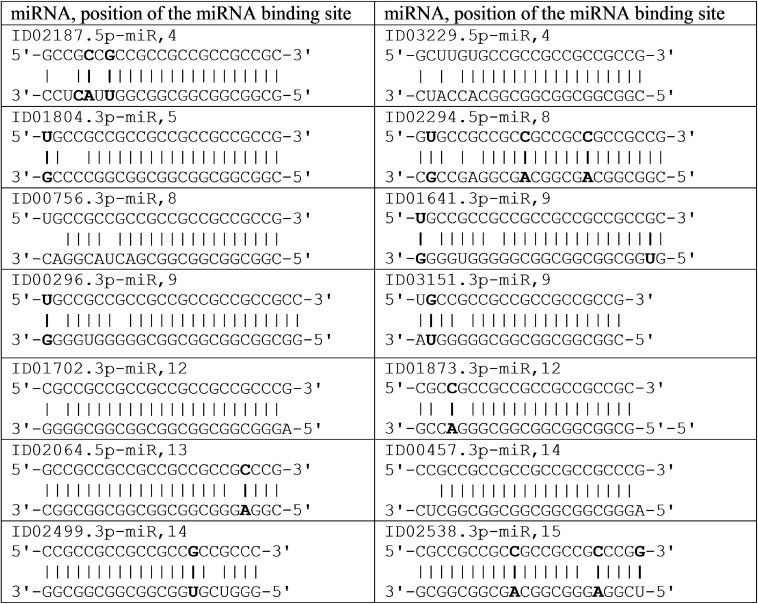
Schemes of miRNA interaction in cluster 5′UTR mRNA of the candidate *GSK3B* gene for Parkinson’s disease.

The efficiency of miRNA binding in a cluster is shown on schemes in Figure 3. Due to the formation of non-canonical A-C and G-U pairs, the structure of the miRNA-mRNA complex has a double-stranded helix without the formation of “bubbles,” which increases the free binding energy of RNA strands due to stacking interactions.

The mRNA of the *PPARGC1A* gene contains two clusters of miRNA BSs, from 18 to 70 nt and from 135 to 172 nt (Table 1). Both clusters contain the BSs of several miRNAs with multiple BSs; that is, several of their BSs for one miRNA are located sequentially over two to three nucleotides. For example, the start of the miR-574-5p and ID00470.5p-miR have five and eight BSs, respectively, that are located over two nucleotides. In the second cluster for ID01310.3p-miR and ID03332.3p-miR four and five BSs respectively. Gene *PPARGC1A* has two BSs for ID03332.3p-miR and one for ID02761.3p-miR with a ΔG value greater than −130 kJ/mole. The association of the *PPARGC1A* gene with these miRNAs can be used as a marker for the diagnosis of PD.

With weak gene expression (value RPKM is 10), it is highly probable that several miRNAs can strongly suppress the synthesis of the corresponding proteins and have a decisive influence on the manifestation of their function. In addition, the presence of multiple BSs for the miRNAs ID00470.5p-miR, miR-574-5p, and ID01310.3p-miR in the mRNA of certain genes, such as *PPARGC1A*, significantly increases the probability that their expression will be suppressed.

**FIGURE 4:**
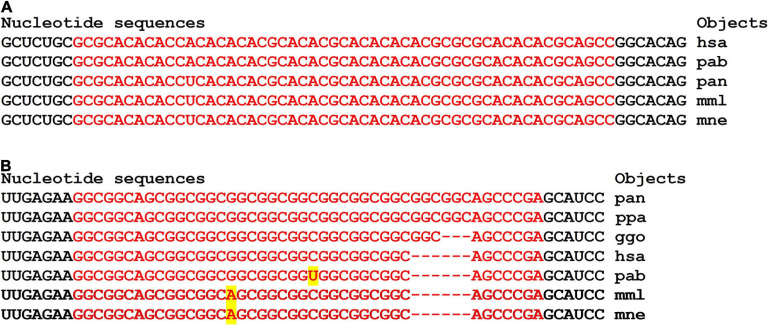
Nucleotide sequences of 5′UTR regions of mRNAs of orthologous *PPARGC1A* genes containing the first cluster **(A)** and the second cluster **(B)** of miRNAs binding sites.

There are clusters of miRNA BS in the mRNA of orthologous genes of monkeys (Figure 4). The first cluster, 53 nt long, is highly conserved (Figure 4A), while the second cluster differs from species to species (Figure 4B).

In the first and second clusters, the flanking oligonucleotides from 5-end (GCUCUGC and UUGAGAA) and 3-end (GGCACAG and GCAUCC) are conserved.

**FIGURE 5:**
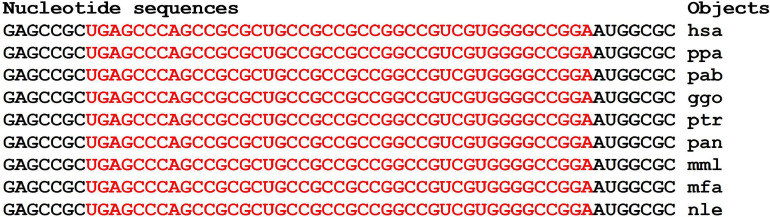
Nucleotide sequences of 5′UTR regions of mRNAs of orthologous ZFAND4 genes containing a cluster of miRNAs binding sites.

There was a cluster in the mRNA of the *ZFAND4* gene containing the BSs for 11 miRNAs (Table 1 and Figure 5). The nucleotide sequences of clusters of mRNA BSs orthologous gene and flanking sequences were highly conserved. Gene *ZFAND4* has miRNA BS for ID00296.3p-miR, ID01190.5p-miR with a ΔG value of more than −130 kJ/mole (Table 1). This association is recommended for use in PD diagnosis.

The miRNA BSs in the mRNA of some genes formed clusters in which these sites featured partially overlapping nucleotides. The mRNA of the *CCNY* gene contained two clusters (Table 1). The first cluster from 1 to 30 nt included the BSs of nine miRNAs with a total length equal to 206 nt, which was 6.9 times greater than the length of the cluster. The organization of the miRNA BSs into clusters has the following consequences. The length of the 5′UTR is 180 nt, and the BSs of nine miRNAs with a length of 206 nt cannot be sequentially located in the 5′UTR without nucleotide overlap. Therefore, compaction of the miRNA BSs is necessary. However, the compaction of the BSs leads to competition between miRNAs for binding to a 30-nt region in which only one miRNA can bind. In this case, miRNA predominantly binds the mRNA with the highest free interaction energy. For example, ID01041.5p-miR, ID00296.3p-miR, ID01702.3p-miR, ID01641.3p-miR, and ID01106.5p-miR preferably bind to the mRNA of the *CCNY* gene. In addition, it must be considered that the concentration of each miRNA can differ by a factor of several tens, and as a result, the quantitative characteristics of the interactions of mRNAs with different miRNAs in combination with their concentrations determines the duration of the miRNA complex with mRNA. For this reason, it is necessary to control the concentration of all miRNAs and mRNAs, which results in the miRNA determining the primary inhibition of translation. The second cluster of BSs for ID02971.3p-miR, ID02128.5p-miR, and ID01976.5p-miR in the mRNA of the *CCNY* gene had a smaller compaction of 1.4-fold. However, in this case, competition also was observed among the three miRNAs for binding to mRNA.

**FIGURE 6:**
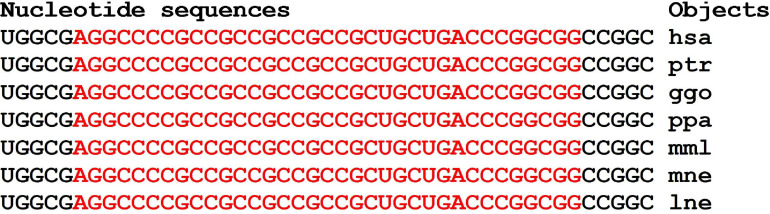
Nucleotide sequences of 5′UTR regions of mRNAs of orthologous *CCNY* genes containing clusters of miRNAs binding sites.

Given in Figure 6, the nucleotide sequences of clusters of BS in the mRNA of the orthologous *CCNY* genes are flanked by conserved oligonucleotides UGGCG and CCGGC.

**FIGURE 7:**
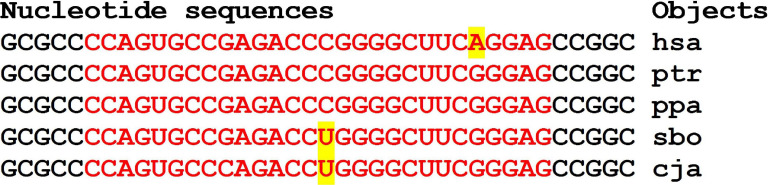
Nucleotide sequences of 5′UTR regions of mRNAs of orthologous *LRP10* genes containing clusters of miRNAs binding sites.

Of the 15 candidate genes with RPKM values less than 10, eleven genes each had a miRNA BSs in the 5′UTR (Supplementary Table 2). The *KANSL1* gene was the potential target of two miRNAs, and the *CRHR1* and *ERBB2* genes were the potential targets of three miRNAs. In the mRNA of the *LRP10* gene, the ID03064.3p-miR and ID01106.5p-miR BSs formed a cluster. The mRNA of the *LRP10* gene contains a cluster from 406 to 434 nt, 28 nt long (Figure 7). The flanking pentanucleotides GCGCC and CCGGC are the same in all objects.

In the mRNA of the *MANF* gene, two miRNAs had sites in the cluster from 56 to 97 nt (Supplementary Table 2). The total length of the BSs of two miRNAs was 69 nt, which was 1.7 times greater than the length of the cluster. Therefore, when organizing BSs into clusters, the sites were compacted to reduce the length of the 5′UTR. Another consequence of this compaction is the emergence of competition among miRNAs for binding to mRNA, since only one miRNAs can interact with a 41-nt-long cluster.

The *RAB5A* gene is the potential target of six miRNAs, the BSs of which form three clusters (Supplementary Table 2). ID03445.3p-miR has two BSs with overlapping nucleotide sequences, which increases the likelihood of its interaction with the mRNA of the *RAB5A* gene. Compared with other miRNAs, the association of ID02930.3p-miR has a large free energy of interaction with the mRNA of the *RAB5A* gene and can be recommended as a marker for PD diagnosis.

In 20 genes with low expression levels, 14 miRNA and mRNA associations were identified (Table 1 and Supplementary Table 2). Six miRNA and mRNA associations were identified in 15 genes with high expression in the 5′UTR mRNA. Fourteen associations between miRNAs [ID00061.3p-miR, ID00296.3p-miR, ID01041.5p-miR, ID01106.5p-miR, ID01190.5p-miR, ID01702.3p-miR, ID01804.3p-miR, ID02064.5p-miR, ID02761.3p-miR, ID03047.3p-miR, ID03064.3p-miR (two sites), and ID03332.3p-miR] and the 5′UTRs of mRNAs of candidate PD target genes (*ERBB2, GSK3B, LRCH1, LRP10, PPARGC1A, ZFAND4*, and *CCNY*) have free energy interactions of more than −130 kJ/mole (Table 1 and Supplementary Table 2) and are recommended as markers for PD.

Of the 15 candidate genes, seven had one BS for different miRNAs (Table 1 and Supplementary Table 2). Each of the *CTNNB1, MANF, MAPT, RTN1*, and *VSNL1* genes were targets of two miRNAs (Supplementary Table 2).

A small number of BSs with the formation of clusters (Supplementary Table 2) is characteristic of most genes with high expression in addition to those shown in Table 1. Only in the mRNA of the *CDK5R1, MART*, and *VSNL1* genes were the clusters of two miRNA BSs identified. The free energy of the interactions of these miRNAs with mRNAs of candidate PD genes was a ΔG value below −130 kJ/mole. In 13 genes with high expression, there were no such associations (Supplementary Table 2).

### Characteristics of the Interactions Between miRNAs and the CDSs of mRNAs of Candidate PD Genes

In the CDSs of the mRNAs of six genes, there was one BS, and in the mRNAs of nine genes, there were two BSs (Supplementary Table 3). Only in the mRNAs of the *AXIN1, CD5*, and *ERBB2* genes were clusters of BSs for two miRNAs detected. The *FOXO1* gene was the potential target of seven miRNAs, the BSs of which were distributed across two clusters (Table 2). From 655 to 695 nt, there were five BSs with a total length of 137 nt, which was 3.3 times greater than the length of this cluster. The value of the free energy of the interactions between the miRNAs and mRNAs for the three associations of miRNAs and mRNAs of *FOXO1* was above −130 kJ/mole.

**TABLE 2:** Characteristics of the interactions between miRNAs and CDS mRNAs of candidate PD genes.

**Gene; RPKM**	**miRNA**	**Start of site, nt**	**ΔG, kJ/mole**	**ΔG/ΔGm, %**	**Length, nt**
*APOE;* 269.2	ID03402.5p-miR	758	−121	95	22
	ID03398.5p-miR	881	−115	93	20
	ID03261.5p-miR	883	−115	93	20
*FOXO1*; 2.5	ID03332.3p-miR	655, 658	−136, −140	91, 94	24
	ID02761.3p-miR	661	−132	89	24
	ID02611.3p-miR	660	−125	91	22
	ID00171.3p-miR	666	−117	93	20
	ID01804.3p-miR	672	−136	93	23
	ID01057.5p-miR	745	−123	91	23
	ID02429.3p-miR	749	−123	91	23
*SETD1A*; 4.5	miR-6824-5p	2,062	−113	90	22
	[0.2] miR-1207-5p	2,064	−115	93	21
	ID00850.3p-miR	2,495	−117	90	22
	ID01321.5p-miR	2,498	−113	91	21
	[19.1] miR-762	4,098	−125	92	22
	miR-6891-3p	4,759	−106	93	21
	ID03324.3p-miR	4,764, 4,788	−115	90	22
	ID03238.3p-miR	4,769	−117	90	23
	ID01545.3p-miR	4,776	−113	93	21
	ID02538.3p-miR	4,877	−121	90	22
	ID01641.3p-miR	4,894, 4,900	−132, −140	89, 94	24
	ID01323.3p-miR	4,898	−123	91	22
	miR-3960	4,899	−115	92	20
	ID00296.3p-miR	4,900	−140	89	25
	ID01702.3p-miR	4,900	−134	89	24
	ID01959.3p-miR	4,905	−117	92	21
	ID00962.3p-miR	4,905	−117	89	23

The nucleotide sequences of mRNAs binding site clusters of orthologous *FOXO1* genes are highly conserved (Figure 7). The high GC-content of miRNAs BS determines the high free energy of miRNAs binding to mRNAs of the *FOXO1* gene. The oligonucleotides flanking the clusters are conserved.

**FIGURE 8:**
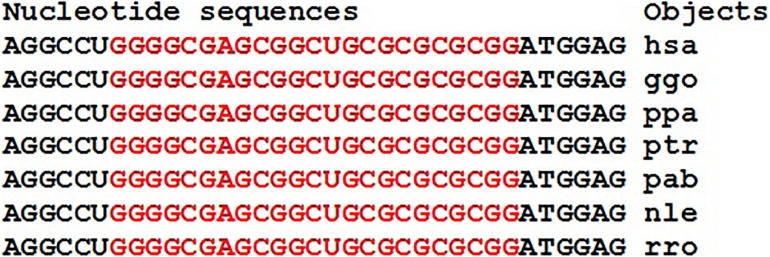
Nucleotide sequences of the CDSs mRNAs regions of orthologous *APOE* genes containing binding sites of ID01030.3p-miR and ID03261.3p-miR.

Clusters of miRNA BSs were identified only in the mRNAs of the orthologous *APOE* genes. The nucleotide sequences of the cluster and the oligonucleotides flanking them were conserved (Table 2 and Figure 8). The protein regions encoded by BS of ID01030.3p-miR and ID03261.3p-miR in mRNA orthologous *APOE* genes were conserved, respectively (Supplementary Figure 1).

**FIGURE 9:**
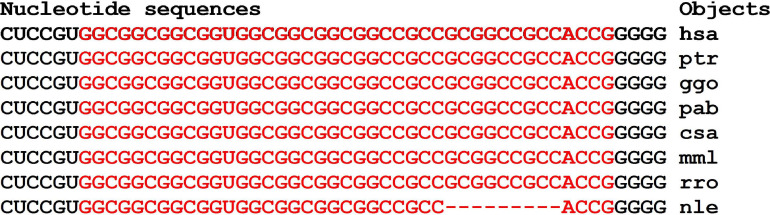
Nucleotide sequences of CDS regions of mRNAs of orthologous *FOXO1* genes containing clusters of miRNAs binding sites.

The miRNA BS in the mRNA of 11 orthologous *FOXO1* genes of some mammals formed clusters encoding longer oligonucleotides (Figure 9). However, the flanking amino acid sequences from the C-terminus were identical and from the N-terminus differed by one amino acid (Supplementary Figure 2).

The clusters of miRNAs BS in mRNAs of the *FOXO1* gene encoded the same oligopeptides AAAVAAAAAAAA, with the exception of the nle-mRNAs of the *FOXO1* gene (Supplementary Figure 2). The amino acid sequences flanking the oligopeptides encoded by the cluster of BS were completely conserved.

The miRNA BS in the mRNA of 11 orthologous *FOXO1* genes of some mammals formed clusters encoding longer oligonucleotides (Supplementary Figure 3). However, the flanking amino acid sequences from the C-terminus were identical and from the N-terminus differed by one amino acid.

Among the associations of miRNAs and mRNA of *FOXO1*, ID02761.3p-miR, ID03332.3p-miR, ID01804.3p-miR stand out, which are recommended as markers of PD as interacting with a ΔG value of more than −130 kJ/mole (Table 2).

**FIGURE 10:**
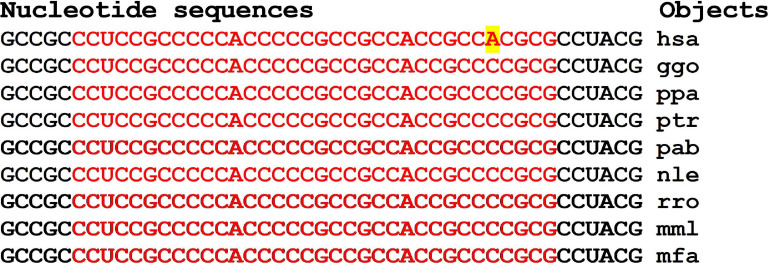
Nucleotide sequences of the CDSs mRNAs regions of orthologous *SETD1A* genes containing clusters of miRNAs BSs.

The mRNA of the *SETD1A* gene contained 17 miRNA BSs (Table 2). ID03324.3p-miR and ID01641.3p-miR each had two BSs in different clusters. The cluster of BSs from 4,877 to 4,928 nt was four times less than the total length of miRNA BSs, which was 205 nt (Figure 10). Highly conserved nucleotide sequences of cluster encode conserved amino acids in orthologous proteins (Supplementary Figure 4). Six associations between miRNAs (ID00296.3p-miR, ID01641.3p-miR, ID01702.3p-miR target genes *SETD1A* have a free energy of the miRNA interaction with the CDS of more than −130 kJ/mole (Table 2) and are recommended as markers for PD. In 34 genes with low expression, only two genes with clusters of BSs had six associations with a ΔG value higher than −130 kJ/mole.

**FIGURE 11:**
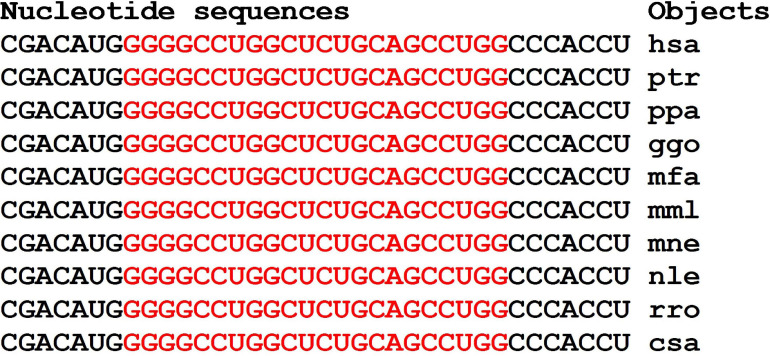
Nucleotide sequences of the CDSs mRNAs regions of orthologous *ATN1* genes containing the ID01047.3p-miR BSs.

Each mRNA of the *ATN1* and *ATP13A2* genes had BSs for seven miRNAs (Supplementary Table 3). Only in the mRNA of the *ATP13A2* gene was a cluster of two BSs for ID01157.5p-miR and ID01377.3p-miR identified. Therefore, in the CDSs of mRNAs of the candidate PD genes, there were no clusters of BSs of more than two miRNAs. BS of ID01047.3p-miR is conserved in the mRNA of *ATN1* orthologous genes (Figure 11). Corresponding amino acid sequences were conserved along with flanking oligopeptides (Supplementary Figure 5). Of the 16 genes with high expression in the protein-encoding region, no miRNA BSs with free interaction energies higher than −130 kJ/mole were found.

### Characteristics of miRNA Interactions With 3′UTRs of mRNAs of Candidate PD Genes

Each of the mRNAs of ten candidate genes bound to only one miRNA (Supplementary Table 4). The mRNAs of the *LRP10, PRKN, RBBP5*, and *SLC14A1* genes were potential targets for two or three miRNAs, containing a cluster of miR-5095 and miR-619-5p BSs located six nucleotides apart (Table 3 and Supplementary Table 4). The mRNA of the *GSK3B* gene, in addition to the miRNA BSs in the 5′UTR, contained miRNA BSs in the 3′UTR (Table 3), which made up the cluster of BSs for miR-466, ID01030.3p-miR, and ID00436.3p-miR, and together with ID01727.5p-miR, these miRNAs could bind to the mRNA of the *PPARGC1A* gene (Table 3).

**TABLE 3:** Characteristics of the interactions between miRNAs and the 3′UTR mRNAs of candidate PD genes.

**Gene; RPKM**	**miRNA**	**Start of site, nt**	**ΔG, kJ/mole**	**ΔG/ΔGm, %**	**Length, nt**
*GSK3B*; 8.3	ID01030.3p-miR	4,705÷4,719 (4)	−108÷−113	89÷93	23
	miR-466	4,709÷4,721 (6)	−104÷−106	89÷91	23
	ID00436.3p-miR	4,713÷4,723 (3)	−104	89	23
	ID01727.5p-miR	4,722	−106	91	23
*LRP10*; 7.8	(1.6) miR-5096	3,237	−104	92	21
	ID02175.3p-miR	3,353	−110	91	22
	[3.6] miR-5585-3p	3,305	−115	98	22
	[5.3] miR-1285-5p	3,404	−102	91	21
	[4.3] miR-619-5p	3,497	−110	91	22
	[19.7] miR-4452	3,544	−108	94	23
	[8.3] miR-5095	3,788	−106	91	21
	[4.3] miR-619-5p	3,794	−115	95	22
	ID00913.5p-miR	3,814	−117	92	23
*PDP2*; 1.2	ID00047.3p-miR	3,220	−110	93	21
	[1.6] miR-5096	3,920	−108	96	21
	[4.3] miR-619-5p	3,980	−113	93	22
	[3.6] miR-5585-3p	3,987	−106	91	22
	[5.3] miR-1285-5p	4,086	−102	91	21
	ID01200.3p-miR	4,511	−102	91	21
	[0.2] miR-1273a	4,639	−119	90	25
	[3.3] miR-1273c	4,641	−110	91	22
	[8.3] miR-1273g-3p	4,661	−106	91	21
	ID01360.3p-miR	5,493	−104	91	21
	miR-3159	5,861	−106	91	22
	[4.3] miR-619-5p	5,863	−113	93	22
	[4.3] miR-619-5p	5,988	−110	91	22
	[4.3] miR-619-5p	6,173	−119	98	22
	[1.6] miR-5096	6,247	−108	96	21
	[4.3] miR-619-5p	6,308	−117	96	22
	ID01836.5p-miR	6,398	−113	90	23
	[1.6] miR-5096	6,413	−102	91	21
*PPARGC1A*; 2.5	miR-466	3,321, 3,337	−106	91	23
	ID00436.3p-miR	3,323÷3,339 (3)	−104÷−108	89÷93	23
	ID01030.3p-miR	3,325	−115	95	23
	ID01727.5p-miR	3,338	−104	89	23
*RBBP5*; 3.9	(8.3) miR-5095	3,065	−108	93	21
	[4.3] miR-619-5p	3,071	−113	93	22
	[1.6] miR-5096	3,145	−106	94	21
	ID03006.5p-miR	4,015	−121	89	24
	[4.3] miR-619-5p	4,030	−115	95	22
	[1.6] miR-5096	4,104	−106	94	21
	miR-3159	4,163	−106	91	22
	ID02175.3p-miR	4,220	−113	93	22
	ID01237.3p-miR	4,271	−117	92	24
*SLC14A1*; 8.5	(8.3) miR-5095	2,771	−110	95	21
	[4.3] miR-619-5p	2,777	−119	98	22
	[1.6] miR-5096	2,851	−102	91	21
	[4.3] miR-619-5p	3,215	−115	95	22
	ID01836.5p-miR	3,003	−113	90	23
*VSNL1*; 206.5	(1.5) miR-574-5p	1,021÷1,045 (13)	−108 ÷−113	89–93	23
	ID00470.5p-miR	1,023÷1,045 (12)	−108	89	23

**FIGURE 12:**
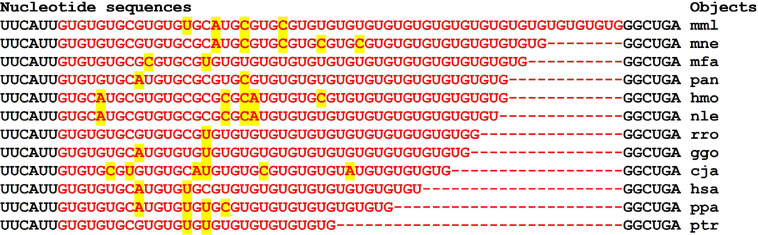
Nucleotide sequences of 3′UTR regions of mRNAs of orthologous *GSK3B* genes containing clusters of miRNAs BSs.

The data shown in Figure 12 indicate a difference in the size of the cluster of miRNAs BS in the 3′UTR of mRNA of *GSK3B* orthologous genes. At the same time, the flanking oligonucleotides remain highly conserved. These results prove the emergence of a connection between miRNA and mRNA of target genes many millions of years ago. The organization of miRNA BS into clusters also has a long history. The existing changes in the nucleotide composition of BS occur according to the principle of replacement of purine for purine (A↔G), or pyrimidine for pyrimidine (U↔C). Such substitutions result in non-canonical pairs G-U and A-C. MirTarget takes these interactions into account and predicts the formation of these pairs.

Note that the preservation of the oligonucleotide composition of the cluster-flanking BS in the 3′UTR of orthologous genes during evolution is probably necessary to preserve the interactions of miRNAs with mRNAs. Note that the flanking nucleotides contain the same CUUGGU hexanucleotides (Supplementary Figure 7).

**FIGURE 13:**
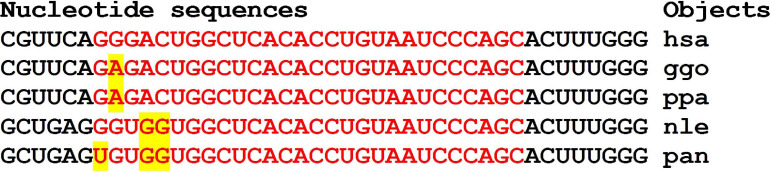
Nucleotide sequences of 3′UTR regions of mRNAs of orthologous *SLC14A1* genes containing clusters of miR-5095 and miR-619-5p BSs.

The *LRP10* gene is the potential target of nine miRNAs. The miR-5095 and miR-619-5p BSs form a cluster, and the beginnings of their BSs differed by six nucleotides. This relationship between miR-5095 and miR-619-5p BSs is not accidental, since the identical arrangement of their BSs was determined in the mRNAs of the *PRKN* (Supplementary Table 4), *RBBP5*, and *SLC14A1* genes (Table 3 and Figure 13).

In addition, the difference between the miR-5096 and miR-619-5p BSs was the same (74 nt) in the mRNAs of the *PDP2, RBBP5*, and *SLC14A1* genes. The beginnings of the miR-5585-3p and miR-1285-5p BSs differed by 99 nt in the mRNAs of the *LRP10* and *PDP2* genes. The miR-619-5p bound to the mRNA of the *PRKN* gene fully complementarily among the 201 genes that are the target of this miRNA (Atambayeva et al., 2017). Candidate PD genes that are targets of miRNAs that bind to the 3′UTRs of mRNAs significantly differ from other candidate genes in the number of BSs for miR-619-5p, miR-5095, miR-5096, miR-5585-3p, and miR-1285-5p. Another feature of candidate PD genes is the association of the *GSK3B* and *PPARGC1A* genes with miR-466, ID00436.3p-miR, ID01030.3p-miR, and ID01727.5p-miR, the BSs of which form one cluster (Table 3). The interactions between miRNAs and the 3′UTRs of mRNAs occur with less free energy than those between miRNAs and the 5′UTRs and CDSs of mRNAs because these miRNAs have reduced GC contents. For example, only ID02732.3p-miR was associated with the mRNA of the *PRKN* gene, exhibiting a ΔG value of −132 kJ/mole.

**FIGURE 14:**
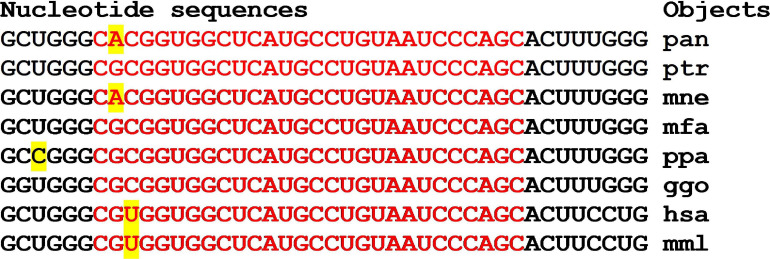
Nucleotide sequences of 3′UTR regions of mRNAs of orthologous *PRKN* genes containing clusters of binding sites miR-5095 and miR-619-5p.

The cluster of miR-5095 and miR-619-5p BS in the mRNA of the *SLC14A1* gene is conserved in part of miR-619-5p binding (GGCUCACACCUGUAAUCCCAGC) and is variable in the six nucleotide segment that binds to miR-5095 (Figure 13). Flanking nucleotides from the 3-end of the cluster are the same for all objects (ACUUUGGG) and coincide with the flanking nucleotides of the 3-end of the cluster in the mRNA of the *PRKN* gene of most objects (Figure 14).

The schemes of miRNA and mRNA nucleotide interactions are a clear illustration of the effectiveness of the MirTarget program in establishing miRNA BSs in mRNA of candidate PD genes (Supplementary Figure 6). These schemes demonstrate the important role of non-canonical U and G, A and C pairs in maintaining the double-stranded structure of the miRNA-mRNA complex while maintaining the stacking interaction between miRNA and mRNA nucleotides, which gives their complex increased stability.

**FIGURE 15:**
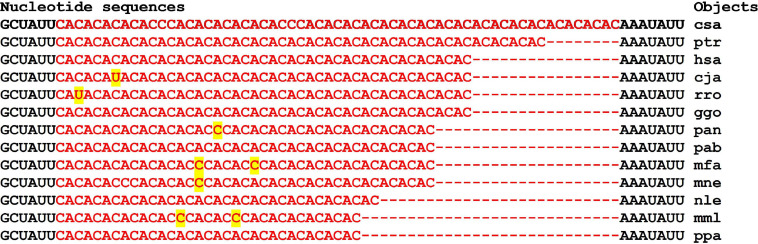
Nucleotide sequences of 3′UTR regions of mRNAs of orthologous *VSNL1* genes containing clusters of miRNAs binding sites.

In 21 genes with high and low expression, no miRNA-miRNA associations with a ΔG value of more than −130 kJ/mole were found. However, miRNA associations with multiple BSs in the mRNA of candidate target genes can be proposed as associations for diagnostics. These miRNAs and the target gene may include miR-574-5p, ID00470.5p-miR, and *VSNL1* (Table 3 and Figure 15).

Of the large family of miR-1273a,c,d,e,f,g-5p or -3p (Ivashchenko et al., 2014a) only a few candidate genes have been targeted by some miR-1273 (Table 3). A cluster of BS miR-1273a, miR-1273c, and miR-1273g-3p with an efficiency of their binding ΔG/ΔGm of more than 90% was detected in the mRNA of the hsa-PDP2 gene. For mRNA of ptr*-PDP2*, ppa*-PDP2*, and ggo*-PDP2* orthologous genes, the ΔG/ΔGm value was 85% or more. mRNA of orthologous genes pab*-PDP2*, mfa*-PDP2*, mml-*PDP2*, mne-*PDP2*, mmu*-PDP2*, rno*-PDP2* interacted with miR-1273a, miR-1273c, and miR-1273g-3p with a ΔG/ΔGm value of less than 80%, which indicates a weak interaction of these miRNA and mRNA. Diagrams of the corresponding associations for hsa, ggo, ppa, ptr are shown in Supplementary Figure 8 and demonstrate the interaction of miRNA and mRNA without bubbles. Note that in the clusters of BS, purine for purine and pyrimidine for pyrimidine is replaced, which insignificantly affects the free energy of interaction between miRNA and mRNA. Oligonucleotides before and after the cluster of BS miR-1273a, miR-1273c, and miR-1273g-3p were conserved (Table 3), which reflects the need to maintain the position of the cluster of BS for these miRNAs. Based on the results presented, the association of miR-1273a, miR-1273c, miR-1273g-3p, and the PDP2 gene can be proposed as a marker for the diagnosis of PD.

The mRNA of the *CCNY* gene, in addition to the 5′UTR BSs, had BSs for six miRNAs in the 3′UTR (Supplementary Table 4). The miR-1273a, ID03224.5p-miR, and miR-1273g-3p BSs formed a cluster 45 nt long with a total length of 69 nt BSs. The mRNA of the *DIRAS1* gene had two clusters of miRNA BSs that started at 929 and at 3,443 nt. This placement of clusters led to the competition of miRNA in each of the clusters for binding to the mRNA of the *DIRAS1* gene (Supplementary Table 4). Consequently, the highly expressed *CCNY*, *DIRAS1*, and *VSNL1* genes have clusters of miRNA BSs in their mRNAs. In the mRNA of the *WDR82* gene, a cluster of miR-5095 and miR-619-5p BSs was detected with a difference of six nucleotides in the start sites of the BSs. Candidate PD genes with high expression levels did not have miRNA BSs with free energy greater than −130 kJ/mole in the 3′UTRs of their mRNAs (Table 3 and Supplementary Table 4).

Note that genes expressed with RPKM values from 0.1 to 10 had an average RPKM value of 3.5 ± 2.9 and the host genes of intronic miRNAs had an average RPKM value of 4.6 ± 2.8 (Supplementary Table 5). The correlation coefficient between the RPKM values of the host genes and 51 target genes of their miRNA was equal to 0.26, that is, there was no strong relationship between the expression of miRNA and potential target genes.

The expression of miRNA and the expression of their target genes were comparable, which indicates the need to maintain close concentrations of miRNA and corresponding mRNA in the norm.

For target genes with a high RPKM value of 43.1–322.5 (average value 116.8), the RPKM value of miRNA host genes varied in the range 1.2–22.4 (average value 7.8). Therefore, these miRNAs normally only slightly alter the expression of target genes, since the expected miRNA concentration will be about 15 times less than the mRNA concentration. However, in pathology, the concentration of miRNA can increase tens of times, or the expression of the gene can decrease many times, and then their significant interaction will occur. This analysis should be taken into account when interpreting the experimental results of determining the concentrations of miRNA and mRNA target genes that make up the association for the diagnosis of the disease. Most of the miRNAs that act on candidate genes for PD are new miRNAs. Unfortunately, we have no information about which of them are of intronic origin. However, the significant similarity between the properties of old and new miRNAs (Aisina et al., 2019; Kondybayeva et al., 2020; Mukushkina et al., 2020) allows us to hope that the relationships revealed in this work between the expression of old miRNAs and their potential target genes are also characteristic of new miRNAs.

## Discussion

Our studies have shown that for many known PD candidate genes, their mRNAs are effectively targeted by miRNAs. The *in silico* characteristics of the interactions between miRNAs and mRNAs can be used in calculating the inhibition efficiency of the translation process at different ratios of miRNA and mRNA concentrations. Thus, using the kinetic equations of the analysis of the interaction of the inhibitor and the enzyme, we can interpret the effect of miRNA by changing the ratio of the mRNA and miRNA concentrations.

The correlations established in many published reports between the changes in the concentration of one or several miRNAs and the changes in the expression of putative target genes involved in the development of PD are not very reliable. This lack of reliability is observed because in most studies of PD and other diseases, the concentration of miRNA was not controlled simultaneous to the expression of the putative target genes. The results of their interactions strongly depend on the ratio of the miRNA concentrations and mRNA concentrations of the target genes. For example, even with strong binding of miRNA to mRNA, the suppression of gene expression is negligible if the concentration of miRNA is significantly lower than the concentration of mRNA. Conversely, with an average interaction of miRNA with mRNA and an excess of the miRNA concentration over the mRNA concentration, strong translation inhibition is observed. Therefore, in the tables, we also present low characteristics of the binding of miRNAs to mRNAs of candidate genes. These associations of miRNAs and genes can be markers with increasing concentrations of miRNAs relative to mRNAs. Considering the competition between miRNAs upon binding to mRNAs, the problem of establishing an effective miRNA for a particular gene becomes even more complicated, since the expression of several or even tens of miRNAs and genes needs to be controlled. Bioinformatics approaches make it possible to select from several thousand miRNAs that are likely to interact with candidate PD genes, which significantly reduces the material costs of searching for miRNAs and target gene associations.

Based on the results obtained in this study, the following generalizations can be made. Not all of the more than 200 candidate PD genes were targets of miRNA. Out of 6,756 miRNAs, only 150 miRNAs were identified that efficiently bound to the mRNA of 61 candidate PD genes. The miRNA BSs were located in the 5′UTRs, CDSs and 3′UTRs of the mRNAs of candidate PD genes. Each of more than half of the candidate genes was the potential target of one miRNA. The mRNAs of the remaining genes could bind two or more miRNAs. The BSs of most miRNAs were located along the entire length of the mRNA without overlapping nucleotide sequences. In the mRNA of some genes, miRNA BSs located in overlapping nucleotide sequences (clusters) were detected. Such clusters reduced by several times the proportion of nucleotide sequences of miRNA BSs in the 5′UTRs, CDSs and 3′UTRs. The miRNAs with BSs in the cluster compete with one another, and only one of these miRNAs can bind to mRNA. The miRNA with a large free energy of interaction with the mRNA and present in a higher concentration compared to other miRNAs has the advantage for binding. The start of the miR-619-5p and miR-5095 BSs are located over six nucleotides, thereby forming a cluster. As a rule, the free energy of the interaction of the mRNA with miR-619-5p is greater than that with miR-5095 (Table 3 and Supplementary Table 3). However, if the concentration of miR-5095 is two to three times higher than the concentration of miR-619, then it is more likely to suppress translation. If the cluster contains the BSs of many miRNAs, then more complex calculations are required to establish the miRNAs with the greatest influence on the translation process.

In the CDSs of mRNAs of almost all low and highly expressed genes, there were miRNA BSs that were not repeated in other genes (Table 2 and Supplementary Table 3). In other words, these associations of miRNA and candidate PD target genes are specific, which gives them preference for use in diagnosis. A feature of some PD candidate genes is the presence of clusters containing BSs for the same set of miRNAs in their mRNAs (Tables 1, 3 and Supplementary Table 4). Differences in the binding characteristics of miRNAs to the mRNAs of genes expressed at different rates have been established. Multiple BSs of miR-466, ID01030.3p-miR, ID00436.3p-miR, and ID01727.5p-miR were located in the 3′UTRs of the mRNAs of the *PPARGC1A* and *GSK3B* genes with low expression (Table 3). The miR-5095 and miR-619-5p BSs formed a cluster, and the beginnings of their BSs differed by six nucleotides. This connection of miR-5095 and miR-619-5p BSs is not random, since it is observed in the mRNA of the *LRP10, PDP2, PRKN, RBBP5, SLC14A1*, and *WDR82* genes (Table 3 and Supplementary Table 4). In addition, the difference between the miR-5096 and miR-619-5p BSs (74 nt) was the same in the mRNAs of the *PDP2, RBBP5*, and *SLC14A1* genes. The start of the miR-5585-3p and miR-1285-5p BSs differed by 99 nt in the mRNA of the *LRP10* and *PDP2* genes. miR-619-5p binds to the mRNA of the *PRKN* gene completely complementarily among 201 genes that are the potential target of this miRNA (Atambayeva et al., 2017). Candidate PD genes that are targets of miRNAs that bind in 3′UTRs of mRNAs significantly differed from other candidate genes by the number of miR-619-5p, miR-5095, miR-5096, miR-5585-3p, and miR-1285-5p BSs (Table 3 and Supplementary Table 4; Ivashchenko et al., 2014a).

In the 5′UTRs of the mRNAs of genes, miRNA BSs were more frequently organized into clusters (Table 1). The identified features of the interactions between miRNAs and the mRNAs of candidate PD genes should be taken into account when selecting miRNA associations and potential target genes for diagnosing the disease.

Based on the quantitative characteristics of the interactions between miRNAs and mRNAs, the associations of miRNAs and candidate genes with a high free energy of interaction were identified, which are recommended as markers for the diagnosis of PD. For the diagnosis of diseases, it is preferable to use miRNA associations with BS in the 5′UTRs of candidate genes, since the free energy of interaction between miRNAs and mRNAs has a higher value than in CDSs and 3′UTRs (Tables 1–3). Let us consider examples of the association of miRNAs and genes on which the development of PD can depend to a greater extent. Clusters of ID00296.3p-miR and ID01702.3p-miR BSs were detected in the mRNAs of the *GSK3B, SETD1A*, and *CCNY* genes. Therefore, it is necessary to control the expression of both miRNAs and the three genes to evaluate the role of these associations in the development of the disease. This approach is necessary in elucidating the role of other associations of miRNAs and genes in the development of PD. For example, the association of several miRNAs (ID01041.5p-miR, ID00457.3p-miR, ID03367.5p-miR, and ID02770.5p-miR) and the *GSK3B* gene shows the need to control the binding of these miRNAs in two clusters in the mRNA of the *GSK3B* gene (Table 1). In addition to the two considered examples of miRNAs and gene associations, other associations will be considered below, which generally demonstrate the need to control a large number of miRNAs and candidate gene expression levels. There is no other approach to determine which miRNAs out of the currently known 6,266 miRNAs can regulate the development of PD. The bioinformatics approach enables only dozens of effective associations to be selected from many millions of associations between miRNAs and mRNAs.

In the 5′UTR of the mRNA of the *PPARGC1A* gene, there was a cluster of ID00470.5p-miR and miR-574-5p BSs, each of which had five sequentially located BSs (Table 1). In the 3′UTR of the mRNA of the *VSNL1* gene, these miRNAs had more than ten multiple BSs (Table 3). Therefore, these miRNAs can have a greater effect on the expression of these genes than miRNAs with one BS. With point mutations of nucleotides (e.g., single nucleotide polymorphisms) in a cluster with multiple BSs, the effectiveness of these miRNAs does not change substantially.

Analysis of the role of the expression of candidate genes in the form of potential miRNA targets leads to the following conclusions. It is expected that for the regulation of highly expressed genes, comparably high concentrations of miRNAs are required; otherwise, if the miRNAs are present at lower concentrations than the mRNAs, they will not significantly regulate the translation process. Based on the above considerations, the concentrations of miRNA and mRNA should be comparable. Therefore, there is a conserved relationship between the nucleotide sequences of miRNAs and miRNA BSs in the mRNA (Davis et al., 2005; Wang et al., 2016; Yurikova et al., 2019). While recommending the association of miRNAs and potential target genes for disease diagnosis, we emphasize that miRNA and mRNA concentrations must be simultaneously recorded. Without these quantitative indicators, it is difficult to draw conclusions regarding the significance of the data obtained.

## Data Availability Statement

The original contributions presented in the study are included in the article/Supplementary Material, further inquiries can be directed to the corresponding author/s.

## Author Contributions

AI and AAr conceived of the study, drafted the manuscript, and gave final approval of the version to be published. SK and AK conceived of the study and drafted the manuscript. AAk analyzed the effect of miR-619-5p and miR-5095 on genes, conceived of the study, and drafted the manuscript. All authors made substantial contributions to acquisition of data, to interpretation and modification of the data, were involved in subsequent rounds of revisions, and read and approved the final manuscript.

## Conflict of Interest

The authors declare that the research was conducted in the absence of any commercial or financial relationships that could be construed as a potential conflict of interest.

## Footnotes

1 http://www.ncbi.nlm.nih.gov

2 https://www.proteinatlas.org/ENSG00000150093-ITGB1/tissue/
