# Supplementary material for: Evolutionary Changes in the Interaction of miRNA With mRNA of Candidate Genes for Parkinson’s Disease
Source: Front Genet. 2021 Mar 30;12:647288. doi: 10.3389/fgene.2021.647288 (PMC8042338; doi:10.3389/fgene.2021.647288)
Supplement: Supplementary file 1 [file Table_1.DOCX]

**Supplementary Table S1** The database of 61 candidate genes responsible for the development of PD

| Gene | ID | PMID | Gene | ID | PMID |
| --- | --- | --- | --- | --- | --- |
| *APOE* | 348 | 31505070 | *MAPT* | 4137 | 32961270 |
| *APP* | 351 | 30745444 | *MYL4* | 4635 | 29936662 |
| *ATN1* | 1822 | 24534762 | *PDP2* | 57546 | 29936662 |
| *ATP13A2* | 23400 | 31588715 | *PINK1* | 65018 | 31540955 |
| *AXIN1* | 8312 | 30537735 | *PLA2G6* | 8398 | 31496990 |
| *BCL2* | 596 | 30508559 | *PPARGC1A* | 10891 | 30236862 |
| *BOLA2* | 552900 | 28586827 | *PRKN* | 5071 | 31409571 |
| *CASK* | 8573 | 29852216 | *PSEN1* | 5663 | 29692703 |
| *CCNY* | 219771 | 26253177 | *PSEN2* | 5664 | 29692703 |
| *CD5* | 921 | 30871733 | *PSMD6* | 9861 | 29852216 |
| *CDK5R1* | 8851 | 21130530 | *RAB5A* | 5868 | 31138985 |
| *CRHR1* | 1394 | 28586827 | *RBBP5* | 5929 | 25187168 |
| *CTNNB1* | 1499 | 27692691 | *RTN1* | 6252 | 28623007 |
| *DIRAS1* | 148252 | 29936662 | *SETD1A* | 9739 | 28586827 |
| *DYRK1A* | 1859 | 29698690 | *SLC14A1* | 6563 | 28623007 |
| *EEF1A1* | 1915 | 29852216 | *SMOX* | 54498 | 22761592 |
| *EIF4G1* | 1981 | 30598256 | *SNAP25* | 6616 | 30334187 |
| *ERBB2* | 2064 | 15857400 | *SNCA* | 6622 | 31686421 |
| *FOXO1* | 2308 | 22761592 | *SRMS* | 6725 | 29936662 |
| *GAK* | 2580 | 28586827 | *STIP1* | 10963 | 30335591 |
| *GBA1* | 2629 | 31729779 | *STK32B* | 55351 | 29899728 |
| *GCH1* | 2643 | 30245141 | *SYMPK* | 8189 | 28586827 |
| *GRN* | 2896 | 29692703 | *SMOX* | 54498 | 22761592 |
| *GSK3B* | 2932 | 25829335 | *TH* | 7054 | 32977678 |
| *HSP90AA1* | 3320 | 30584159 | *TPO* | 7173 | 31891753 |
| *KANSL1* | 284058 | 32829096 | *VPS35* | 55737 | 32853677 |
| *LAG3* | 3902 | 31847878 | *VSNL1* | 7447 | 28623007 |
| *LRCH1* | 23143 | - 29936662 | *UBL4B* | 164153 | 29936662 |
| *LRP6* | 4040 | 28341812 | *WNT3* | 7473 | 31504236 |
| *LRP10* | 26020 | 32409252 | *ZFAND4* | 93550 | 29936662 |
| *MANF* | 7873 | 29959908 |  |  |  |
