# Supplementary material for: Evolutionary Changes in the Interaction of miRNA With mRNA of Candidate Genes for Parkinson’s Disease
Source: Front Genet. 2021 Mar 30;12:647288. doi: 10.3389/fgene.2021.647288 (PMC8042338; doi:10.3389/fgene.2021.647288)
Supplement: Supplementary file 2 [file Table_2.DOCX]

**Supplementary Table S2** Characteristics of miRNA interactions with 5′UTR mRNA of candidate PD genes

| Gene; RPKM | miRNA | Start of  site, nt | ΔG,  kJ/mole | ΔG/ΔGm,  % | Length,  nt |
| --- | --- | --- | --- | --- | --- |
| *AXIN1*; 6.0 | ID02769.5p-miR | 214 | -125 | 91 | 22 |
| *BOLA2*; 0.6 | miR-4783-3p | 130 | -123 | 91 | 23 |
| *CD5*; 0.1 | miR-5585-3p | 55 | -106 | 91 | 22 |
| *CRHR1*; 5.8 | ID01291.3p-miR | 69 | -129 | 90 | 24 |
|  | ID01291.3p-miR | 107 | -127 | 88 | 24 |
|  | ID00331.5p-miR | 179 | -127 | 88 | 24 |
| *DYRK1A*; 7.3 | ID03444.3p-miR | 40 | -104 | 89 | 23 |
| *ERBB2*; 3.2 | ID00529.5p-miR | 21 | -108 | 91 | 21 |
|  | ID03064.3p-miR | 122 | -136 | 89 | 24 |
|  | ID01206.3p-miR | 165 | -127 | 90 | 23 |
| *FOXO1*; 2.5 | ID00951.5p-miR | 120 | -110 | 91 | 21 |
|  | ID00661.5p-miR | 228 | -110 | 91 | 22 |
|  | ID00009.3p-miR | 305 | -115 | 92 | 20 |
|  | ID02200.3p-miR | 356 | -119 | 90 | 22 |
|  | miR-4728-3p | 363 | -125 | 88 | 25 |
| *KANSL1*; 6.3 | ID00130.3p-miR | 153 | -119 | 89 | 24 |
|  | miR-6846-3p | 391 | -106 | 93 | 21 |
| *LAG3*; 0.3 | ID02025.5p-miR | 241 | -104 | 91 | 21 |
| *LRCH1*; 1.8 | ID03047.3p-miR | 201 | -132 | 89 | 24 |
| *LRP6*; 2.4 | miR-6752-5p | 69 | -119 | 90 | 22 |
| *LRP10*; 7.8 | miR-1229-3p | 272 | -115 | 89 | 23 |
|  | ID03064.3p-miR | 406 | -138 | 90 | 24 |
|  | ID01106.5p-miR | 410 | -132 | 89 | 24 |
|  | ID00462.3p-miR | 480 | -115 | 90 | 22 |
| *MANF*; 7.1 | ID00278.3p-miR | 56 | -123 | 89 | 23 |
|  | ID01282.3p-miR | 59 | -117 | 89 | 23 |
|  | ID01768.3p-miR | 67 | -115 | 92 | 22 |
|  | ID01352.3p-miR | 71, 74 | -115, -117 | 90, 92 | 23 |
|  | ID00777.3p-miR | 75, 78 | -113 | 90 | 23 |
|  | ID03324.3p-miR | 76 | -115 | 90 | 22 |
| *PRKN*; 3.4 | miR-6861-5p | 108 | -117 | 93 | 22 |
| *PSEN1*; 4.7 | ID00895.3p-miR | 119 | -117 | 89 | 23 |
| *PSEN2*; 4.6 | ID02019.3p-miR | 10 | -125 | 89 | 23 |
| *ZFAND4*; 0.5 | ID01190.5p-miR | 114 | -136 | 89 | 24 |
|  | ID00030.3p-miR | 114 | -125 | 94 | 22 |
|  | ID03206.5p-miR | 114 | -115 | 92 | 20 |
|  | ID03073.3p-miR | 128 | -129 | 94 | 23 |
|  | ID02215.5p-miR | 407 | -115 | 89 | 23 |
| *ATN1*; 83.0 | ID01919.5p-miR | 178 | -108 | 89 | 23 |
| *ATP13A2*; 49.4 | ID00338.3p-miR | 101 | -117 | 92 | 21 |
| *CDK5R1*; 41.3 | ID03224.5p-miR | 33 | -123 | 94 | 23 |
|  | miR-1273g-3p | 46 | -110 | 95 | 21 |
|  | miR-1273f | 79 | -98 | 94 | 19 |
| *CTNNB1*; 27.7 | ID00477.5p-miR | 77 | -113 | 95 | 20 |
|  | ID03206.5p-miR | 152 | -115 | 92 | 20 |
| *EIF4G1*; 24.7 | ID01208.5p-miR | 186 | -115 | 89 | 23 |
| *MAPT*; 38.4 | ID02608.5p-miR | 107 | -113 | 90 | 22 |
|  | ID01315.3p-miR | 120 | -115 | 92 | 20 |
| *PINK1*; 63.5 | ID00553.3p-miR | 30 | -117 | 89 | 23 |
| *PSMD6*; 20.0 | miR-6510-5p | 113 | -110 | 90 | 22 |
| *RTN1*; 187.1 | ID01545.3p-miR | 5 | -110 | 91 | 21 |
|  | ID01310.3p-miR | 67 | -125 | 95 | 22 |
| *SMOX*; 18.0 | ID01319.5p-miR | 13 | -106 | 93 | 20 |
|  | ID00443.5p-miR | 40 | -123 | 89 | 24 |
|  | ID00570.3p-miR | 98 | -113 | 90 | 22 |
| *SNAP25*; 563.3 | ID03210.5p-miR | 212 | -127 | 90 | 24 |
| *STIP1*; 30.1 | ID01628.5p-miR | 375 | -117 | 93 | 20 |
| *VSNL1*; 206.5 | miR-7111-3p | 52 | -115 | 95 | 22 |
|  | miR-877-3p | 52 | -108 | 93 | 21 |
