# Supplementary material for: Evolutionary Changes in the Interaction of miRNA With mRNA of Candidate Genes for Parkinson’s Disease
Source: Front Genet. 2021 Mar 30;12:647288. doi: 10.3389/fgene.2021.647288 (PMC8042338; doi:10.3389/fgene.2021.647288)
Supplement: Supplementary file 3 [file Table_3.DOCX]

**Supplementary Table S3** Characteristics of miRNA interactions with CDS mRNAs of candidate PD genes

| Gene; RPKM | miRNA | Start of  site, nt | ΔG,  kJ/mole | ΔG/ΔGm,  % | Length,  nt |
| --- | --- | --- | --- | --- | --- |
| *ASH2L*; 10.7 | ID03311.5p-miR | 158 | -108 | 93 | 21 |
| *AXIN1*; 6.0 | ID02727.5p-miR | 2479 | -115 | 92 | 21 |
|  | ID01279.5p-miR | 2484 | -110 | 90 | 22 |
| *BCL2-al*; 2.8 | miR-1343-5p | 606 | -121 | 90 | 22 |
|  | ID02256.3p-miR | 715 | -125 | 91 | 22 |
| *CASK*; 2.8 | ID00526.3p-miR | 291 | -115 | 89 | 24 |
|  | miR-6746-3p | 2680 | -115 | 90 | 22 |
| *CD5*; 0.1 | ID00446.3p-miR | 159 | -119 | 90 | 23 |
|  | miR-6721-5p | 522 | -115 | 92 | 23 |
|  | miR-6793-5p | 543 | -110 | 91 | 22 |
|  | miR-6791-3p | 740 | -108 | 91 | 21 |
|  | ID01761.3p-miR | 1573 | -102 | 91 | 21 |
| *CRHR1*; 5.8 | ID02323.3p-miR | 969 | -115 | 90 | 22 |
| *ERBB2*; 3.2 | ID00692.3p-miR | 3646 | -113 | 90 | 22 |
|  | miR-4734 | 3648 | -119 | 90 | 22 |
| *GAK*; 14.4 | miR-3689d | 2018 | -110 | 91 | 22 |
|  | ID02646.3p-miR | 3007 | -113 | 95 | 20 |
|  | ID01021.3p-miR | 3019 | -115 | 90 | 22 |
|  | ID01048.5p-miR | 3059 | -117 | 89 | 23 |
| *GRN*; 11.9 | let-7g-3p | 846 | -106 | 91 | 21 |
| *KANSL1*; 6.3 | miR-302b-3p | 2075 | -104 | 91 | 23 |
|  | miR-4706 | 3701 | -123 | 87 | 25 |
| *LAG3*; 0.3 | ID01184.3p-miR | 560 | -119 | 95 | 20 |
|  | ID00920.5p-miR | 611 | -125 | 88 | 24 |
|  | ID01542.3p-miR | 711 | -119 | 89 | 23 |
| *LRP6*; 2.4 | ID03063.3p-miR | 3421 | -110 | 90 | 22 |
| *LRP10*; 7.8 | miR-1229-3p | 1856 | -115 | 89 | 23 |
| *MYL4*; 0.1 | miR-4763-3p | 189 | -129 | 91 | 24 |
| *PDP2*; 1.2 | miR-802 | 1963 | -104 | 91 | 23 |
|  | ID01038.3p-miR | 1723 | -106 | 91 | 21 |
| *PLA2G6*; 5.9 | miR-3619-5p | 881 | -117 | 93 | 22 |
|  | ID02257.3p-miR | 1861 | -117 | 90 | 22 |
| *SMOX*; 18.0 | miR-5047 | 593 | -102 | 91 | 21 |
|  | ID00211.3p-miR | 1093 | -113 | 90 | 22 |
|  | ID03343.3p-miR | 1101 | -110 | 90 | 22 |
|  | miR-3148 | 1240 | -102 | 91 | 22 |
| *SRMS*; 0.1 | ID00879.3p-miR | 486 | -123 | 89 | 23 |
|  | miR-6733-3p | 853 | -100 | 96 | 20 |
| *STK32B*; 0.6 | miR-520g-5p | 582, 975 | -104 | 89 | 23 |
| *SYMPK*; 12.7 | miR-762 | 3619 | -125 | 92 | 22 |
|  | ID00744.3p-miR | 3626 | -125 | 89 | 23 |
| *TPO*; 0.1 | ID02309.3p-miR | 1089 | -121 | 89 | 23 |
|  | ID01446.5p-miR | 1152 | -119 | 92 | 22 |
| *WNT3*; 2.7 | ID02756.3p-miR | 615 | -117 | 89 | 23 |
| *APOE; 269.2* | ID03402.5p-miR | 758 | -121 | 95 | 22 |
|  | ID03398.5p-miR | 881 | -115 | 93 | 20 |
|  | ID03261.5p-miR | 883 | -115 | 93 | 20 |
| *APP; 186.8* | ID01171.3p-miR | 2404 | -104 | 92 | 20 |
| *CTNNB1; 27.7* | miR-6083 | 1773 | -98 | 92 | 20 |
| *EEF1A1; 111.6* | ID02627.5p-miR | 1150 | -117 | 96 | 22 |
| *EIF4G1; 24.7* | ID02781.3p-miR | 623 | -115 | 92 | 20 |
|  | ID03020.3p-miR | 4758 | -117 | 89 | 23 |
| *HSP90AA1; 322.5* | miR-3613-5p | 1770 | -93 | 90 | 22 |
|  | ID02619.5p-miR | 2578 | -102 | 91 | 22 |
| *MAPT; 38.4* | ID01128.5p-miR | 744 | -110 | 90 | 23 |
|  | miR-7106-5p | 1008 | -106 | 94 | 20 |
| *RTN1; 187.1* | ID01172.3p-miR | 1426 | -110 | 91 | 21 |
|  | ID00194.3p-miR | 1617 | -115 | 89 | 23 |
|  | ID03137.5p-miR | 1931 | -115 | 92 | 20 |
| *SNCA; 39.2* | miR-4668-3p | 352 | -100 | 89 | 23 |
|  | miR-4677-5p | 427 | -110 | 98 | 22 |
