# Supplementary material for: Evolutionary Changes in the Interaction of miRNA With mRNA of Candidate Genes for Parkinson’s Disease
Source: Front Genet. 2021 Mar 30;12:647288. doi: 10.3389/fgene.2021.647288 (PMC8042338; doi:10.3389/fgene.2021.647288)
Supplement: Supplementary file 4 [file Table_4.DOCX]

**Supplementary Table S4** Characteristics of miRNA interactions with 3′UTR mRNA of candidate PD genes with RPKM

| Gene; RPKM | miRNA | | Start of  site, nt | ΔG,  kJ/mole | ΔG/ΔGm,  % | Length,  nt |
| --- | --- | --- | --- | --- | --- | --- |
| *BCL2-alpha*; 2.8 | ID03441.3p-miR | | 3613 | -110 | 90 | 22 |
| *CCNY*; 19.7 | ID02833.5p-miR | | 1591 | -121 | 90 | 22 |
|  | miR-1273a | | 4336 | -117 | 89 | 25 |
|  | ID03224.5p-miR | | 4346 | -119 | 90 | 23 |
|  | miR-1273g-3p | | 4359 | -106 | 91 | 21 |
|  | miR-1273e | | 4402 | -108 | 93 | 22 |
|  | miR-1972 | | 4597 | -113 | 91 | 22 |
| *CD5*; 0.1 | ID00845.3p-miR | | 2578 | -121 | 90 | 23 |
| *CRHR1b*; 5.8 | miR-1202 | | 1707 | -110 | 91 | 21 |
|  | miR-1914-5p | | 1800 | -115 | 90 | 22 |
|  | miR-516b-5p | | 2497 | -102 | 91 | 22 |
| *DIRAS1*; 57.9 | miR-1228-5p | | 929 | -115 | 93 | 21 |
|  | ID00121.5p-miR | | 931 | -110 | 96 | 18 |
|  | ID02992.5p-miR | | 932 | -110 | 96 | 18 |
|  | ID02339.5p-miR | | 1033 | -119 | 90 | 23 |
|  | ID00850.3p-miR | | 3443 | -117 | 90 | 22 |
|  | ID01382.3p-miR | | 3443 | -113 | 93 | 20 |
| *FOXO1*; 2.5 | miR-6754-5p | | 4453 | -113 | 91 | 22 |
|  | ID02346.3p-miR | | 4489 | -93 | 92 | 21 |
| *GBA1*; 6.9 | miR-6826-3p | | 2517 | -108 | 91 | 22 |
| *GCH1*; 1.1 | ID02289.5p-miR | | 2526 | -98 | 92 | 22 |
| *LRCH1*; 1.8 | ID00649.3p-miR | | 4860 | -102 | 91 | 21 |
| *LRP6*; 2.4 | miR-1277-5p | | 6096, 6098 | -96 | 88 | 24 |
|  | miR-4693-3p | | 9160 | -108 | 94 | 23 |
| *PRKN*; 3.4 | ID02732.3p-miR | | 1613÷1617(3) | -121÷ -132 | 89÷97 | 23 |
|  | ID00470.5p-miR | | 1620 | -108 | 89 | 23 |
|  | miR-574-5p -miR | | 2614 | -104 | 91 | 22 |
|  | ID02783.5p-miR | | 3310 | -98 | 92 | 20 |
|  | miR-5095 | | 3723 | -110 | 95 | 21 |
|  | miR-619-5p | | 3729 | -121 | 100 | 22 |
|  | ID02175.3p-miR | | 3917 | -110 | 91 | 22 |
|  | miR-1285-5p | | 3968 | -110 | 98 | 21 |
| *RBBP5*; 3.9 | miR-5095 | | 3065 | -108 | 93 | 21 |
|  | miR-619-5p | | 3071 | -113 | 93 | 22 |
|  | miR-5096 | | 3145 | -106 | 94 | 21 |
|  | ID03006.5p-miR | | 4015 | -121 | 89 | 24 |
|  | miR-619-5p | | 4030 | -115 | 95 | 22 |
|  | miR-5096 | | 4104 | -106 | 94 | 21 |
|  | miR-3159 | | 4163 | -106 | 91 | 22 |
|  | ID02175.3p-miR | | 4220 | -113 | 93 | 22 |
|  | ID01237.3p-miR | | 4271 | -117 | 92 | 24 |
| *SRMS*; 0.0 | miR-574-5p | | 2142 | -115 | 95 | 23 |
| *TH*; 0.3 | ID00091.5p-miR | | 1697 | -117 | 89 | 23 |
|  | miR-149-3p | | 1836 | -113 | 91 | 21 |
| *UBL4B*; 0.2 | miR-4639-3p | | 1160 | -100 | 94 | 20 |
| *ATN1*; 83.0 | ID00759.5p-miR | 3924 | | -110 | 93 | 21 |
| *EEF1A1*; 111.6 | ID01404.5p-miR | 2726 | | -115 | 93 | 23 |
|  | miR-1273e | 2732 | | -110 | 95 | 22 |
|  | miR-1972 | 2911 | | -113 | 91 | 22 |
| *MAPT*; 38.4 | ID03372.3p-miR | 3298 | | -106 | 93 | 21 |
|  | miR-650 | 3495 | | -110 | 93 | 21 |
|  | miR-4298 | 4066 | | -113 | 90 | 22 |
| *SMOX*;18.0 | miR-423-5p | 2280 | | -113 | 90 | 23 |
| *VPS35*; 12.6 | miR-5585-3p | 4802 | | -106 | 91 | 22 |
