# Supplementary material for: Evolutionary Changes in the Interaction of miRNA With mRNA of Candidate Genes for Parkinson’s Disease
Source: Front Genet. 2021 Mar 30;12:647288. doi: 10.3389/fgene.2021.647288 (PMC8042338; doi:10.3389/fgene.2021.647288)
Supplement: Supplementary file 5 [file Table_5.DOCX]

**Supplementary Table S5** Characteristics of miRNA’s host gene RPKM level

| miRNA | Host gene | RPKM |
| --- | --- | --- |
| miR-877-3p | *ABCF1*-in-noncod | 10.4 |
| miR-1285-5p | *AC000120*-KRIT1-in-noncod | 5.3 |
| miR-802 | *AF015720*-RUNX1-in-noncod | 0.2 |
| miR-6721-5p | *AGPAT1*- in-noncod | 31.4 |
| miR-6752-5p | *AIP*-in-noncod | 29.5 |
| miR-5096 | *BMP2K*-in-noncod | 1.6 |
| miR-423-5p | *CCDC55*-in-noncod | 5.2 |
| miR-5047 | *DDX5*-in-ex-noncod-cod | 57.6 |
| miR-6733-3p | *EBNA1BP2*- in-noncod | 14.4 |
| miR-4728-3p | *ERBB2*-in-noncod | 3.2 |
| miR-6746-3p | *FADS3*-in-noncod | 22.7 |
| miR-574-5p | *FAM114A1*-in-noncod | 1.5 |
| miR-4706 | *FNTB*-in-noncod | 5.8 |
| miR-149-3p | *GPC1*-in-noncod | 42.1 |
| miR-6083 | *KALRN*-in-noncod | 9.4 |
| miR-6510-5p | *KRT15*-in-noncod | 0.1 |
| miR-1228-5p | *LRP1*-in-noncod | 21.2 |
| miR-4298 | *LSP1*-in-noncod | 0.5 |
| miR-4452 | *MAPK10*-in-noncod | 19.7 |
| miR-1229-3p | *MGAT4B*-in-noncod | 12.3 |
| miR-4639-3p | *MYLIP*-in-noncod | 4.3 |
| miR-6754-5p | *NADSYN1*-in-noncod | 3.3 |
| miR-1972 | *PDXDC1*-in-noncod | 8.6 |
| miR-4783-3p | *PROC*-in-noncod | 0.2 |
| miR-1207-5p | *PVT1*-in-noncod | 0.2 |
| miR-1273a | *RGS22*-in-noncod | 0.2 |
| miR-4693-3p | *RP11*-in-noncod | 19.1 |
| miR-762 | *RP11*-in-noncod | 19.1 |
| miR-4763-3p | *RP4*- in-noncod | 0.3 |
| miR-3619-5p | *RP4*-in-noncod | 0.3 |
| miR-1273f | *SCP2*-in-noncod | 8.3 |
| miR-1273g-3p | *SCP2*-in-noncod | 8.3 |
| miR-5095 | *SCP2*-in-noncod | 8.3 |
| miR-4677-5p | *SDCCAG8*-in-noncod | 4.3 |
| miR-619-5p | *SSH1*-in-noncod | 4.3 |
| miR-1273c | *TIAM2*-in-noncod | 3.3 |
| miR-5585-3p | *TMEM39B*-in-noncod | 3.6 |
| miR-3613-5p | *TRIM13*-in-noncod | 2.5 |
| miR-1914-5p | *UCKL1*-in-noncod | 8.3 |
| miR-4668-3p | *UGCG*-in-noncod | 4.9 |
| miR-1277-5p | *WDR44*-in-noncod | 3.4 |
| let-7g-3p | *WDR82*-in-noncod | 25.1 |
| miR-1273e | unknown |  |
| miR-1343-5p | unknown |  |
| miR-3960 | unknown |  |
| miR-302b-3p | unknown |  |
| miR-650 | unknown |  |
| miR-1202 | intergenic |  |
| miR-466 | intergenic |  |
| miR-516b-5p | intergenic |  |
| miR-520g-5p | intergenic |  |
| miR-6791-3p | intergenic |  |
| miR-6793-5p | intergenic |  |
| miR-6824-5p | intergenic |  |
| miR-6826-3p | intergenic |  |
| miR-6846-3p | intergenic |  |
| miR-6861-5p | intergenic |  |
| miR-6891-3p | intergenic |  |
| miR-7106-5p | intergenic |  |
| miR-7111-3p | intergenic |  |
| miR-3148 | intergenic |  |
| miR-3159 | intergenic |  |
| miR-4734 | intergenic |  |
