# Supplementary material for: Evolutionary Changes in the Interaction of miRNA With mRNA of Candidate Genes for Parkinson’s Disease
Source: Front Genet. 2021 Mar 30;12:647288. doi: 10.3389/fgene.2021.647288 (PMC8042338; doi:10.3389/fgene.2021.647288)
Supplement: Supplementary file 6 [file Image_1.pdf]

Amino acid sequences	Object
ERAQAWGERLRARMEEMGS	hsa
ERAQAWGERLRARMEEMGS	ggo
ERAQAWGERLRARMEEMGS	ppa
ERAQAWGERLRARMEEMGS	ptr
ERAQAWGERLRARMEEMGS	pab
ERAQAWGERLRARMEEMGS	nle
ERAQAWGERLRARMEEMGS	rro

Figure S1 Protein regions encoded by binding sites of ID01030.3p-miR and ID03261.3p-miR in mRNA orthologous *APOE* genes.
