# Supplementary material for: Evolutionary Changes in the Interaction of miRNA With mRNA of Candidate Genes for Parkinson’s Disease
Source: Front Genet. 2021 Mar 30;12:647288. doi: 10.3389/fgene.2021.647288 (PMC8042338; doi:10.3389/fgene.2021.647288)
Supplement: Supplementary file 7 [file Image_2.pdf]

Amino acid sequences	Object
DFPQAPGSVAAAVAAAAAAAAATGGLCGDFQG	hsa
DFPQAPGSVAAAVAAAAAAAAATGGLCGDFQG	ptr
DFPQAPGSVAAAVAAAAAAAAATGGLCGDFQG	ggo
DFPQAPGSVAAAVAAAAAAAAATGGLCGDFQG	pab
DFPQAPGSVAAAVAAAAAAAAATGGLCGDFQG	csa
DFPQAPGSVAAAVAAAAAAAAATGGLCGDFQG	mml
DFPQAPGSVAAAVAAAAAAAAATGGLCGDFQG	rro
DFPQAPGSVAAAVAAAAA---TGGLCGDFQG	nle

Figure S2 Protein regions encoded by clusters of miRNA binding sites in mRNA orthologous *FOXO1* genes.
