# Supplementary material for: Evolutionary Changes in the Interaction of miRNA With mRNA of Candidate Genes for Parkinson’s Disease
Source: Front Genet. 2021 Mar 30;12:647288. doi: 10.3389/fgene.2021.647288 (PMC8042338; doi:10.3389/fgene.2021.647288)
Supplement: Supplementary file 8 [file Image_3.pdf]

```
Amino acid sequences                        Object
DFQQAPGSVAAAAAAAAAAVAAAAAAAAAAATGGLCGDFQG  chi
DFQQAPGSVAAAAAAAAA-VAAAAAAAAAAATGGLCGDFQG  oar
DFQQAPGSVAAAAAAAAA-VAAAAAAAAAA-TGGLCGDFQG  bbu
DFQQAPGSVAAAAAAAAA-VAAAAAAAAA--TGGLCGDFQG  ord
DFQQAPGSVAAAAAAAAA-VAAAAAAAAA--TGGLCGDFQG  ppr
DFQQAPGSVAAAAAAAAA-VAAAAAAAAA--TGGLCGDFQG  uah
DFQQAPGSVAAAAAAAAA-VAAAAAAAAA--TGGLCGDFQG  fca
DFQQAPGSVAAAAAAAA--VAAAAAAAAA--TGGLCGDFQG  oor
DFQQAPGSVAAAAAAAA--VAAAAAAAAA--TGGLCGDFQG  dle
DFQQAPGSVAAAAAAAAA-VAAAAAAAA---TGGLCGDFQG  bta
DFQQAPGSVAAAAAAAAA-VAAAAAAAA---TGGLCGDFQG  ssc
```

Figure S3 Protein regions encoded by clusters of miRNA binding sites in mRNA orthologous *FOXO1* genes.
