# Supplementary material for: Evolutionary Changes in the Interaction of miRNA With mRNA of Candidate Genes for Parkinson’s Disease
Source: Front Genet. 2021 Mar 30;12:647288. doi: 10.3389/fgene.2021.647288 (PMC8042338; doi:10.3389/fgene.2021.647288)
Supplement: Supplementary file 9 [file Image_4.pdf]

Amino acid sequences	Objects
HARRRRPPPPPPPPPPRAYEPRS	hsa
HARRRRPPPPPPPPPPRAYEPRS	ggo
HARRRRPPPPPPPPPPRAYEPRS	ppa
HARRRRPPPPPPPPPPRAYEPRS	ptr
HARRRRPPPPPPPPPPRAYEPRS	pab
HARRRRPPPPPPPPPPRAYEPRS	nle
HARRRRPPPPPPPRPPRAYEPRS	rro
HARRRRPPPPPPPRPPRAYEPRS	mml
HARRRRPPPPPPPRPPRAYEPRS	mfa

Figure S4 Protein regions encoded by clusters of miRNA binding sites in mRNA of orthologous *SETD1A* genes.
