# Supplementary material for: Evolutionary Changes in the Interaction of miRNA With mRNA of Candidate Genes for Parkinson’s Disease
Source: Front Genet. 2021 Mar 30;12:647288. doi: 10.3389/fgene.2021.647288 (PMC8042338; doi:10.3389/fgene.2021.647288)
Supplement: Supplementary file 10 [file Image_5.pdf]

| Amino acids sequences | Objects |
| --- | --- |
| DPFPRHGGLALQPGPPGLHP | hsa |
| DPFPRHGGLALQPGPPGLHP | ptr |
| DPFPRHGGLALQPGPPGLHP | ppa |
| DPFPRHGGLALQPGPPGLHP | ggo |
| DPFPRHGGLALQPGPPGLHP | mfa |
| DPFPRHGGLALQPGPPGLHP | mml |
| DPFPRHGGLALQPGPPGLHP | mne |
| DPFPRHGGLALQPGPPGLHP | rro |
| DPFPRHGGLALQPGPPGLHP | csa |

**Figure S5** Protein regions encoded by ID01047.3p-miR BSs in the mRNA of orthologous *ATN1* genes of some mammals.
