# Supplementary material for: Evolutionary Changes in the Interaction of miRNA With mRNA of Candidate Genes for Parkinson’s Disease
Source: Front Genet. 2021 Mar 30;12:647288. doi: 10.3389/fgene.2021.647288 (PMC8042338; doi:10.3389/fgene.2021.647288)
Supplement: Supplementary file 11 [file Image_6.pdf]

Gene; miRNA; start of binding site, nt; the miRNA region; the free energy, ΔG, kJ/mole; the ΔG/ΔGm, %; length of miRNA, nt.

PLA2G6,ID02217.5p-miR,107,5'UTR,-134,100,23

```
5'-CCGGACUCCCAAGUCUCCGCCGG-3'
   |||||||||||||||||||||||
3'-GGCCUGAGGGUUCAGAGGCGGCC-5'
```

PPARGC1A,ID00436.3p-miR,3339,3'UTR,-108,93,23

```
5'-GUGUGUGUGUAUGUAUGUGUGUG-3'
   |||||||||||||||||||||||
3'-CACACACGCAUAUAUACACACAU-5'
```

PPARGC1A,ID00470.5p-miR,35,5'UTR,-110,91,23

```
5'-ACGCACACGCACACACACGCGCG-3'
   |||||||||||||||||||||||
3'-UGUGUGUGCGUAUGUGUGCAUGU-5'
```

PRKN,ID00470.5p-miR,1620,3'UTR,-108,89,23

```
5'-ACACACACGCGCGCGCGCGCACA-3'
   |||||||||||||||||||||||
3'-UGUGUGUGCGUAUGUGUGCAUGU-5'
```

SRMS,ID01989.5p-miR,980,CDS,-121,88,23

```
5'-CCUGGGCACCCCCGAGGGCCGGG-3'
   |||||||||| ||||||||||||
3'-GGACCCGCAGUGACCCCCGGCCC-5'
```

GRHL3,ID00920.5p-miR,666,CDS,-123,87,24

```
5'-GCUCCCCUCCCUGCAGGCCCCAGC-3'
   ||||||||||||||||||||||||
3'-CGAAGGGGAGGACGCCCGGGACCG-5'
```

Gene; miRNA; start of binding site, nt; the miRNA region; the free energy, ΔG, kJ/mole; the ΔG/ΔGm, %; length of miRNA, nt.

PPARGC1A,ID00436.3p-miR,3325,3'UTR,-106,91,23

```
5'-GUGUGUGCAUGUAUGUGUGUGUG-3'
   |||||||||||||||||||||||
3'-CACACACGCAUAUAUACACACAU-5'
```

PPARGC1A,ID00470.5p-miR,29,5'UTR,-110,91,23

```
5'-ACACACACGCACACGCACACACA-3'
   |||||||||||||||||||||||
3'-UGUGUGUGCGUAUGUGUGCAUGU-5'
```

PPARGC1A,ID03332.3p-miR,143,5'UTR,-134,90,24

```
5'-GGCGGCGGCGGCGGCGGCGGCGGC-3'
   ||||| ||||| |||||||||| |
3'-CCGCCUCCGCCUCCGCCGCCGCGG-5'
```

SRMS,ID00879.3p-miR,486,CDS,-123,89,23

```
5'-AGCGAGAGCAGCCUCGGGGGCUA-3'
   ||||||||||||| |||||||||
3'-UCGCUCCCGUCGGCGCCCCCGGU-5'
```

ATP13A2,ID00182.5p-miR,2321,CDS,-115,89,23

```
5'-CCACUGUGCCCAGCCUGGAGGCA-3'
    ||||| |||||||||||||||
3'-UGUGACUCGGGUCAGACCCCCGU-5'
```

MANF,ID02047.5p-miR,68,5'UTR,-119,89,22

```
5'-CGGCAGCGGAGGAGGAGGAGGA-3'
   |||| |||||||||||||||||
3'-GCCGGCGCCUCCCCCUCCCCCU-5'
```

Figure S6 Schemes and characteristics of the interaction of miRNA with mRNA of candidate PD genes
