# Supplementary material for: Evolutionary Changes in the Interaction of miRNA With mRNA of Candidate Genes for Parkinson’s Disease
Source: Front Genet. 2021 Mar 30;12:647288. doi: 10.3389/fgene.2021.647288 (PMC8042338; doi:10.3389/fgene.2021.647288)
Supplement: Supplementary file 12 [file Image_7.pdf]

Nucleotide sequences	Objects
CUUGGUGAUGGAGUGUGCGUGUGUGCAUGUAUGUGUGUGUAUGUAUGUGUGUGGUG-GUGUGCUUGGU	hsa
CUUGGUGAUGGAGUGUGCGUGUGUGCAUGUAUGUGUGUGUAUGUAUGUGUAUGGUGUGUGUGCUUGGU	pan
CUUGGUGAUGGAGUGUGCGUGUGUGCAUGUAUGUGUGUGUAUGUAUGUGUGUGGUGUGUGUGCUUGGU	pab
CUUGGUGAUGGAGUGUGCGUGUGUGCAUGUAUGUGUGUGU--GUAUGUGUAUGGUGUGUGUGCUUGGU	nle
CUUGGUGAUGCAGUGUGCGUGUGUGCAUGUAUGUGUGUGU--GUAUGUGUAUGGUGUGUGUGCUUGGU	mml
CUUGGUGAUGGAGUGUGCGUGUGUGCAUGUAUGUGUGUAU--GUAUGUGUGUGGUGUGUGUGCUUGGU	ggo
CUUGGUGAUAGAGUGUGCAUGUGUGCAUGUAUGUGUGUAU--GUAUGUGUGUGGUGUGUGUGCUUGGU	ppa
CUUGGUGAUAGAGUGUGCGUGUGUGCAUGUAUGUGUGUAU--GUAUGUGUGUGGUGUGUGUGCUUGGU	ptr

Figure S7 Nucleotide sequences of 3'UTR regions of mRNAs of orthologous *PPARGC1A* genes containing clusters of miRNAs binding sites
