# Supplementary material for: Evolutionary Changes in the Interaction of miRNA With mRNA of Candidate Genes for Parkinson’s Disease
Source: Front Genet. 2021 Mar 30;12:647288. doi: 10.3389/fgene.2021.647288 (PMC8042338; doi:10.3389/fgene.2021.647288)
Supplement: Supplementary file 13 [file Image_8.pdf]

Nucleotide sequences	Objects
UUUUUGUUGUUUUUUGAGACAAGGUCUUGCUUUGUCGCCCAUGCUGGAGUGCAGUGGCAUCAUUAUAACUCAC	hsa
UUUUUGUUGUUUUUUGAAACAAGGUCUUGCUUUGUCGCCCAUGCUGGAGUGCAGUGGCAUCAUUAUAACUCAC	ggo
UUUUUGUUGUUUUUUGAGACAAGGUCUUGCUUCGUCACCCAUGCUGGAGUGCAGUGGCAUCAUUAUAACUCAC	ppa
UUUUUGUUGUUUUUUGAGACAAGGUCUUGCUUCGUCACCCAUGCUGGAGUGCAGUGGCAUCAUUAUAACUCAC	ptr

Figure S8 Nucleotide sequences of 3'UTR regions of mRNAs of orthologous *PDP2* genes containing clusters of miRNAs binding sites
